# Structure and expression of *GSL1* and *GSL2* genes encoding gibberellin stimulated-like proteins in diploid and highly heterozygous tetraploid potato reveals their highly conserved and essential status

**DOI:** 10.1186/1471-2164-15-2

**Published:** 2014-01-02

**Authors:** Sathiyamoorthy Meiyalaghan, Susan J Thomson, Mark WEJ Fiers, Philippa J Barrell, Julie M Latimer, Sara Mohan, E Eirian Jones, Anthony J Conner, Jeanne ME Jacobs

**Affiliations:** 1 The New Zealand Institute for Plant & Food Research Limited, Private Bag 4704, Christchurch 8140, New Zealand; 2 Faculty of Agriculture and Life Sciences, Lincoln University, P.O. Box 84, Canterbury 7647, New Zealand; 3 AgResearch Ltd, Grasslands Research Centre, Private Bag 11008, Palmerston North 4442, New Zealand; 4 Current address: VIB Center for the Biology of Disease, KU Leuven, Herestraat 49, 3000, Leuven, Belgium

**Keywords:** *GSL1*, *GSL2*, Gibberellin stimulated-like proteins, Potato, Snakin, Genome analysis, Transcriptional expression

## Abstract

**Background:**

GSL1 and GSL2, Gibberellin Stimulated-Like proteins (also known as Snakin-1 and Snakin-2), are cysteine-rich peptides from potato (*Solanum tuberosum* L.) with antimicrobial properties. Similar peptides in other species have been implicated in diverse biological processes and are hypothesised to play a role in several aspects of plant development, plant responses to biotic or abiotic stress through their participation in hormone crosstalk, and redox homeostasis. To help resolve the biological roles of GSL1 and GSL2 peptides we have undertaken an in depth analysis of the structure and expression of these genes in potato.

**Results:**

We have characterised the full length genes for both *GSL1* (chromosome 4) and *GSL2* (chromosome 1) from diploid and tetraploid potato using the reference genome sequence of potato, coupled with further next generation sequencing of four highly heterozygous tetraploid cultivars. The frequency of SNPs in *GSL1* and *GSL2* were very low with only one SNP every 67 and 53 nucleotides in exon regions of *GSL1* and *GSL2,* respectively. Analysis of comprehensive RNA-seq data substantiated the role of specific promoter motifs in transcriptional control of gene expression. Expression analysis based on the frequency of next generation sequence reads established that *GSL2* was expressed at a higher level than *GSL1* in 30 out of 32 tissue and treatment libraries. Furthermore, both the *GSL1* and *GSL2* genes exhibited constitutive expression that was not up regulated in response to biotic or abiotic stresses, hormone treatments or wounding. Potato transformation with antisense knock-down expression cassettes failed to recover viable plants.

**Conclusions:**

The potato *GSL1* and *GSL2* genes are very highly conserved suggesting they contribute to an important biological function. The known antimicrobial activity of the GSL proteins, coupled with the FPKM analysis from RNA-seq data, implies that both genes contribute to the constitutive defence barriers in potatoes. The lethality of antisense knock-down expression of *GSL1* and *GSL2*, coupled with the rare incidence of SNPs in these genes, suggests an essential role for this gene family. These features are consistent with the GSL protein family playing a role in several aspects of plant development in addition to plant defence against biotic stresses.

## Background

The gibberellin stimulated-like proteins GSL1 (also known as Snakin-1) and GSL2 (also known as Snakin-2) are cysteine-rich peptides from potato (*Solanum tuberosum* L.) with *in vitro* antimicrobial activity against a wide range of bacteria and fungi [1-5], as well as nematodes [6]. The spectrum of antimicrobial activity is almost identical for GSL1 and GSL2 [2,3]. GSL1 and GSL2 induce rapid aggregation of both Gram-negative and Gram-positive bacteria, and although this response does not correlate with antimicrobial activity, it is still considered that these proteins may play an *in vivo* role in controlling pathogen migration [1-3,5].

Amino acid sequence alignment of GSL1 and GSL2 show similarity with the GAST1 (gibberellic acid stimulated transcript) from tomato [7] and the GASA family (gibberellic acid stimulated in arabidopsis) from arabidopsis [8] and similar members from a wide range of dicotyledonous and monocotyledonous species [9-14]. Based on a limited similarity in amino acid sequence to the hemotoxic, desintegrin-like snake venoms, GSL1 and GSL2 were formerly referred to as Snakin-1 and Snakin-2 [2,3]. However, the term Snakin is inappropriate for these plant-based proteins since GSL1 and GSL2 do not share the RGD residues and functional properties of snake venoms responsible for desintegrin action [15].

Both the *StGSL1* and *StGSL2* genes encode polypeptides that have similar structural features with an N-terminal putative signal sequence congruent with a sub-cellular location in the plant cell wall and a cysteine-rich C-terminal domain [2,3]. GSL1 has a signal sequence of 25 amino acid residues, followed by a 63-amino acid mature peptide (6.9 kDa) with 12 highly conserved cysteine residues [2]. GSL2 has a 23 amino acid-residue signal peptide, followed by an intermediate 15-residue acidic peptide, and then a mature peptide (7.0 kDa) of a 66 amino acid basic peptide with the 12 conserved cysteine residues [3]. GSL1 and GSL2 peptides share several features characteristic of all antimicrobial peptides. The cysteine-rich nature of these peptides is critical for the occurrence of disulphide bridges that are important for enhancing the structural stability under diverse stress conditions [16]. A high frequency of charged amino acids appears to play a key role in the activity against microbes [16], along with the amphipathic structure and cationic charge at physiological pH [17-19]. The prediction of GSL1 three-dimensional structure and disulfide bonding pattern revealed two long α-helices stabilized and maintained by six knotted disulfide bonds between specific cysteine residues [20].

Northern analysis in potatoes established that transcripts of *StGSL1* exhibited highest accumulation in stems, shoot apices, young floral buds and petals, with expression also detected in tubers and carpels, but not in roots, stolons, leaves, sepals or stamens [2,3]. Transcripts in leaves were not induced by either abiotic or biotic stress, or chemical treatments such as jasmonic acid, salicylic acid, isonicotinic acid, abscisic acid, gibberellic acid, and indolacetic acid, leading to the conclusion that GSL1 is a component of the constitutive defence barriers, especially of the storage and reproductive organs [2]. Similar studies on *StGSL2* expression detected the highest accumulation of transcripts in tubers, petals and carpels, with expression also in stems, shoot apices, leaves, flower buds and stamens, but not in roots, stolons and sepals [3]. In contrast to *StGSL1*, the *StGSL2* gene was locally up-regulated in leaves by wounding and abscisic acid treatments, responded weakly to salinity stress, while drought stress or treatments with gibberellic acid, chitosan, jasmonic acid, ethylene, benzo(1,2,3)thiadiazole-7-carbothioic acid or *S*-methyl ester had no effect [3]. *StGSL2* expression was also up-regulated upon infection of tubers with the compatible fungus *Botrytis cinerea*, but down-regulated by the bacterial pathogens *Ralstonia solanacearum* and *Dickeya chrysanthemi* (formerly known as *Erwinia chrysanthemi*), resulting in the overall hypothesis that GSL2 is a component of both constitutive and inducible defence barriers to pathogens [3].

Over-expression of the *StGSL1* gene in transgenic potato plants enhances resistance against two important potato pathogens *Pectobacterium carotovorum* subspecies *carotovorum* (formerly known as *Erwinia carotovora*) and *Rhizoctonia solani*[21]. Transgenic wheat plants over-expressing the *Solanum chacoense GSL1* gene exhibited improved resistance to *Blumeria graminis* f.sp. *tritici*[22]. Likewise, over-expression of the tomato *GSL2* gene in tomato enhanced tolerance to *Clavibacter michiganensis* subsp. *michiganensis* that causes bacterial canker and wilt disease [23]. Viral-induced gene silencing of *GSL2* in *Nicotiana benthamiana* increased susceptibility to wilt disease development induced by *C. michiganensis* subsp. *michiganensis*[24]. Similarly, virus-induced gene silencing of *GSL2* in *Capsicum annuum* increased susceptibility to root-knot nematodes (*Meloidogyne* spp.) [6]. A defence role for GSL1 was also suggested from the observation of decreased virulence of GSL1-sensitive mutants of *Dickeya chrysanthemi* (formerly known as *Erwinia chrysanthemi*) to potato tubers [25]. The antimicrobial mechanism of action for GSL peptides is not known, but in contrast to other antimicrobial peptides from plants, GSL1 and GSL2 do not interact with artificial lipid membranes [1]. A cysteine-rich 6.8 kDa orthologue of GSL2 from French bean (*Phaseolus vulgaris*) was demonstrated to tightly bind to a 25 kDa polypeptide of a proline-rich protein family from legumes and thought to function as a two-component chitin-receptor involved in plant-pathogen interactions through antimicrobial activity and/or signalling [26].

There is no consensus on the biological roles of GSL proteins. Given their *in vitro* antimicrobial activity they are often considered to play important roles in the innate defence against invading microorganisms [2,3,6] and/or to be a key determinant during the interaction between plants and pathogens [25,26]. Similar genes in other species have been implicated in diverse biological processes, including: cell division, cell elongation, cell growth, transition to flowering, somatic embryogenesis and signalling pathways [10-12,27-30]. Despite the highly conserved nature of GSL/GASA amino acid sequences, including 12 cysteine residues at the C-terminus that are probably responsible for the protein structure and biochemical activity, the functions and mode of action of GSL/GASA proteins are not completely elucidated [31]. The prevailing view is that GSL/GASA proteins play a role in several aspects of plant development, plant responses to biotic or abiotic stress through their participation in hormone crosstalk, and redox homeostasis [31]. This is supported by partial silencing of *GSL1* in potato using antisense approaches that resulted in plants with reduced height and smaller leaves resulting from reduced cell division, altered leaf metabolism and cell wall composition [32].

To help resolve the biological roles of GSL1 and GSL2 peptides we have undertaken a thorough analysis of the structure and expression of these genes in potato. We have characterised the full length genes for both *GSL1* and *GSL2* from diploid and tetraploid potato using the genome sequence of potato [33], coupled with further next generation sequencing of highly heterozygous tetraploid cultivars. Specific promoter motifs and exon regions are highly conserved among multiple alleles, suggesting their importance for biological function. Analysis of comprehensive transcriptome data substantiates the role of specific promoter motifs in transcriptional control of gene expression. The lethality of antisense knock-down expression suggests the essential role of this gene family in potatoes.

## Results and discussion

### Allelic polymorphism of *GSL* genes in potato

PCR isolation, cloning and sequencing of the coding region from the autotetraploid potato cultivar ‘Iwa’ revealed two alleles (a1 and a2) for the *GSL1* gene (GenBank accessions FJ195646 and FJ195647) and two alleles (b1 and b2) for the *GSL2* gene (GenBank accessions EU848497 and EU848498). From the frequency of clones with the *GSL1* alleles, it is estimated that Iwa has three copies of the a1 allele (15 of 16 clones) and one copy of the a2 allele (1 of 16 clones). Similarly for the *GSL2* gene, Iwa has three copies of the b1 allele (10 of 12 clones) and one copy of the b2 allele (2 of 12 clones). The a1 and a2 alleles of the *GSL1* gene differed by 18 Single Nucleotide Polymorphisms (SNPs) and four indels of 1–7 nucleotides. All of these variant nucleotides were in the introns, except for only three synonymous SNPs in the exons. Similarly, the b1 and b2 alleles of the *GSL2* gene were polymorphic for 19 SNPs and five indels of 1–18 nucleotides, with only four synonymous SNPs all occurring in the third exon of *GSL2*.

**Figure 1:**
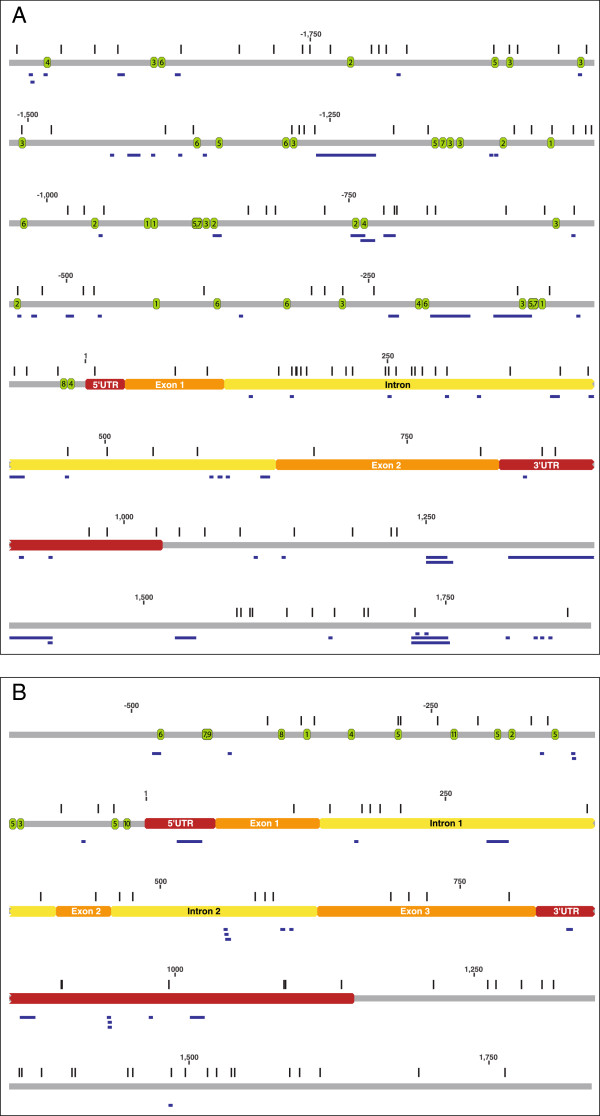
**Schematic representation of the *GSL1 *gene (A) and *GSL2 *gene (B).** The 5’UTR and 3’UTR, exon and intron regions are indicated in red, orange and yellow, respectively. The vertical blue lines indicate positions at which SNPs were observed, the horizontal blue bars are positions where indels occur and the green ovals mark the positions of promoter motifs with the encased numbers indicative of the specific motifs noted in Tables 2 and 3 for the *GSL1* and *GSL2* genes, respectively. This representation is based on a consensus sequence of all genotypes analysed. Due to the presence of polymorphic indels, the exact nucleotide positions do not necessarily match the information for the genotype DM presented in Tables 2 and 3, or Additional file 1: Figure S1 and Additional file 2: Figure S2.

PCR isolation and direct sequencing of cDNA products determined the sequence of cDNA from mature transcripts for both the *GSL1* (GenBank accession GU137307) and *GSL2* (GenBank accession JF683606) genes. Alignment of the genomic and cDNA sequences confirmed the exon and intron regions in both the *GSL1* and *GSL2* genes (Figure 1). The *GSL1* gene consists of two exons of 82 and 187 nucleotides interrupted by a single intron of 525 nucleotides. The *GSL2* gene is composed of three exons of 87, 46 and 182 nucleotides respectively, interspersed with two introns of 268 and 172 nucleotides. The exon/intron boundaries were identical in all alleles of both the *GSL1* and *GSL2* genes, with the splice sites possessing the conserved 5’GT and 3’AG dinucleotides (Additional file 1: Figure S1 and Additional file 2: Figure S2), consistent with the consensus sequences of the intron at both donor and acceptor sites [34].

### Structure of full length *GSL* genes

The sequence of the coding regions of the *GSL1* and *GSL2* genes described above were used for interrogation of the potato genome sequence [33]. Firstly, *GSL1* and *GSL2* nucleotide sequences were used to BLAST search the reference CDS and genomic sequences to identify gene locations on superscaffold assemblies. Secondly, annotated protein sequences from potato genome assembly version 3.4 of the genotype DM1-3 516 R44 (DM) were also searched for the presence of the GASA pfam motif PF02704 (http://pfam.sanger.ac.uk/).

Results showed single copy genes for each of *GSL1* and *GSL2. GSL1* is located on superscaffold PGSC0003DMB000000381 which has been mapped to chromosome 4, whereas *GSL2* is located on superscaffold PGSC0003DMB000000290 which has been mapped to chromosome 1 (Additional file 3: Table S1). Several other genes similar to *GSL* and *GASA* genes were also identified; results of these searches are shown in Additional file 3: Table S1.

The general structure of the *GSL1* and *GSL2* genes was analysed based on the DM sequence of the potato genome [33]. For the *GSL1* gene, up to 1967 nucleotides were recovered upstream from the putative transcription start site. We were only able to confirm up to 616 nucleotides upstream of the putative transcription start site for the *GSL2* gene due to a gap in the assembly of the reference potato genome in the upstream region of the *GSL2* promoter. However, we documented at least 1000 nucleotides downstream of the stop codon for both *GSL* genes. A putative transcription start site was predicted based on a plant dimer motif YR Rule [35] at 33 nucleotides and 38 nucleotides upstream from the first base of the translation start site (ATG) for the *GSL1* and *GSL2* genes, respectively. Putative *cis*-elements were identified for the *GSL1* gene (Additional file 1: Figure S1). These are TATA-box (nucleotides −32 to −27), a pyrimidine patch (Y Patch, nucleotides −26 to −20) and CAAT-box (nucleotides −48 to −44). In *GSL2*, a putative TATA-box and Y Patch were located from nucleotides −50 to −45 and nucleotides −59 to −51, respectively. Since we were unable to locate a satisfactory CAAT-box, we identified a ‘hypothetical’ CAAT-box (nucleotides −65 to −61) in the *GSL2* promoter (Additional file 2: Figure S2).

### Next generation sequencing and SNP discovery

Genomic regions for *GSL1* and *GSL2* were further analyzed for sequence variation by aligning re-sequence data generated from Illumina reads of the diploid RH89-039-16 (RH) [33] and data generated from four tetraploid lines using the Illumina GAIIx platform. Illumina short insert read pair data were generated for each line. Reads were aligned to the reference genome using BWA [36]. Alignments were further analysed using SAMtools [37], polySNP (an in-house developed tool for SNP calling; https://github.com/mfiers/polysnp) and visualized using Geneious [38].

For the diploid RH and the four tetraploid potato genotypes the structure of the *GSL1* and *GSL2* genes and sequence polymorphisms for the various alleles were annotated manually and compared to the reference DM potato genome. The locations of SNPs and indels (insertion/deletions) identified across all alleles from all genotypes is illustrated in Figure 1 and summarized in Table 1. In *GSL1* multiple SNPs and indels were identified within the non-coding regions, with the greatest frequency occurring within the intron and the region from −2000 to −500 nucleotide positions relative to the 5’UTR. The 5’UTR contains only a single SNP that is found in one re-sequenced genotype. The exon regions have no indels and only very rare synonymous SNPs, with an overall SNP frequency in exons of one SNP/67 nucleotides (Table 1, Additional file 4: Table S2). In contrast, the single intron contains 24 SNPs and 16 different indels. The *GSL2* gene also exhibits the highest SNP frequency within the introns (Table 1). A deletion of 21 nucleotides was found within the 5’UTR of DM, but not in the other genotypes. Other indels were found in all gene components except the exons. Consistent with the *GSL1* gene, the exons of *GSL2* exhibit only very rare synonymous SNPs among the alleles from all genotypes, with an overall SNP frequency in exons of one SNP/53 nucleotides (Table 1, Additional file 4: Table S2).

**Table 1:** **The incidence of indels and SNPs in various regions of the *GSL1 *** **(A) and *GSL2 *** **(B) genes**

**Region**	**Nucleotide position**	**Size**	**Number of indels**	**Number of SNPs**	**SNP frequency (nucleotides/SNP)**
**(A)**					
Promoter	−1960 to −1501	460	6	18	25.6
	−1500 to −1001	500	9	14	35.7
	−1000 to −501	500	9	18	27.8
	−500 to −1	500	10	13	38.5
5'UTR	1 to 33	33	0	1	33.0
Exon 1	34 to 115	82	0	2	41.0
Intron	116 to 619	504	16	24	21.0
Exon 2	620 to 804	185	0	2	92.5
3'UTR	805 to 1009	205	3	5	41.0
**(B)**					
Promoter	−590 to −1	590	6	12	49.2
5'UTR	1 to 38	38	1	0	-
Exon 1	39 to 125	87	0	1	87.0
Intron 1	126 to 374	249	2	7	35.6
Exon 2	375 to 420	46	0	1	46.0
Intron 2	421 to 583	163	5	5	32.6
Exon 3	584 to 765	182	0	4	45.5
3'UTR	766 to 1067	302	7	6	50.3

The next generation sequence data from four tetraploid potato genotypes (Karaka, Summer Delight, 1021/1, VT^n^62-33-3), plus the diploid RH [33], were aligned with the reference potato genome of DM [33]. SNP output is from polySNP tool with stringent calling (https://github.com/mfiers/polysnp).

The rare frequency of SNPs observed in *GSL1* and *GSL2* within and between the diploid homozygous DM, the diploid heterozygous RH, and the four tetraploid potato genotypes was comparable to other *GSL* and *GASA*-like genes, as well as other highly conserved housekeeping genes (Additional file 4: Table S2). The rare SNP incidence in these genes across these 19 haplotypes is substantially lower than the one SNP every 29 nucleotides observed in a 6.6 Mb region analyzed for only two RH haplotypes associated with the potato genome sequence [33], and the one SNP every 24 nucleotides (exons) and one SNP every 15 nucleotides (non-coding/introns) reported by Uitdewilligen et al. [39] based on targeted resequencing of 83 tetraploid cultivars. This confirms the highly conserved nature of the *GSL1* and *GSL2* genes, and therefore suggests that they play an essential role in biological function.

### Expression profiles of *GSL* genes

**Figure 2:**
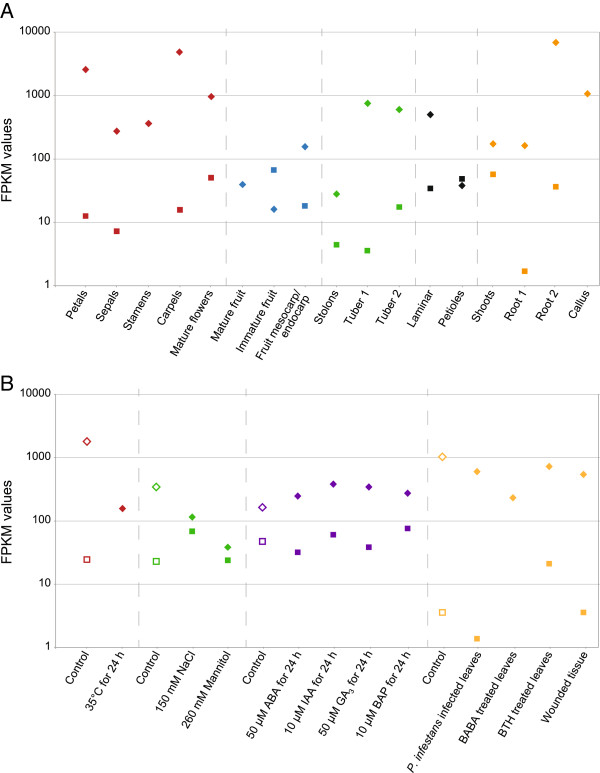
**FPKM values as a representation of transcriptional expression for the potato *GSL1 *and *GSL2 *genes.** Where FPKM values were zero, no data point is graphed. *GSL1* data points are represented by squares and *GSL2* data points are represented by diamonds. **(A)** Different organs of potato DM plant material. **(B)** Stress-related conditions and plant growth regulator treatments using *in vitro* grown potato DM plant material. FPKM values of the controls are represented by an open data point and the treatment values have solid colour.

FPKM (fragments per kilobase per million mapped reads) as expression values for each transcript were extracted from previous data sets [33] representing a range of different potato tissues and treatments (Figure 2). FPKM levels from a total of 32 tissue and treatment libraries from the genotype DM were analyzed for *GSL1* and *GSL2* expression. Of the 32 samples analyzed, *GSL2* was expressed at a higher level in 30 samples compared with *GSL1*, often by over an order of magnitude in FPKM values.

For *GSL1* expression, Segura et al. [2] showed by northern analysis that transcripts of *StGSL1* exhibited highest accumulation in stems, shoot apices, young floral buds and petals. Similarly, FPKM reads showed highest levels in mature flowers, immature fruit, shoots, petals and carpels (Figure 2A). Northern analysis also detected expression in tubers and carpels, but not in roots, stolons, leaves, sepals or stamens [2]. In contrast to the northern analysis, FPKM analysis showed that *GSL1* was expressed in roots, stolons, leaves and sepals. Northern analysis and FPKM data are in agreement for the absence of *GSL1* expression in stamens; with the FPKM data also showing no expression in mature fruit and callus tissue. Northern analysis also established *GSL1* expression is not induced by biotic or abiotic stresses [2], while the FPKM data show an absence of *GSL1* transcripts in response to heat stress or BABA treatments and a slight increase in transcripts in response to BTH treatment (Figure 2B). Analysis of *Arabidopsis thaliana* plants transgenic for GUS fusions to the potato *GSL1* promoter revealed GUS expression in root vascular tissue, cotyledons, young leaves and floral organs [40]. This analysis of transcriptional control by the *GSL1* promoter is more consistent with the FPKM data (Figure 2) than the previously published northern analysis [2].

FPKM values for the *GSL2* gene (Figure 2) and northern analysis [3] are in agreement with highest levels of expression being in carpels and petals, and generally high expression in all tissues examined. However, northern analysis did not detect *GSL2* expression in stolons or roots, which is in contrast to the high levels of expression seen in FPKM data (Figure 2A). In addition, FPKM analysis indicates that *GSL2* expression is not induced by biotic or abiotic stresses, plant growth regulator treatments or wounding (Figure 2B), although slight reductions are observed in *GSL2* transcripts in response to stress induced by heat, salt, mannitol and BABA treatments. This is in contrast with northern analysis, where *GSL2* expression responded to biotic stress, was up-regulated by wounding and ABA treatments, down regulated in response to GA_3_ and showed no response to salinity or drought treatments [3].

The differences between previously published northern analysis for *GSL1*[2] and *GSL2*[3] and the FPKM values in this study (Figure 2) may reflect differences in cultivar/ploidy level and growth/treatment conditions. Overall, the FPKM data support the conclusion that *GSL1* is a component of the constitutive defense barriers, especially of the storage and reproductive organs [2]. The FPKM analysis supports the same conclusion for *GSL2*, which is in contrast to the previous view that *GSL2* is a component of both constitutive and inducible defense barriers [3].

### Analysis of GSL promoters

Since the *GSL1* and *GSL2* genes differed markedly in their magnitude and specificity of transcript accumulation (Figure 2), the promoter regions were analysed for motifs using Genomatix-MatInspector [41] based on PLACE [42]. A total of 58 and 28 different motifs, previously characterised in other studies, were identified in the *GSL1* and *GSL2* promoter regions, respectively (Additional file 5: Table S3 and Additional file 6: Table S4). Based on the known transcriptional expression of the *GSL* genes (Figure 2; [2,3]), the putative roles of GSL proteins [31,32], the repeated occurrence of motifs, their presence in all potato genotypes, and their relative position in the promoter region, key motifs with potential functional significance were identified for *GSL1* (Table 2) and *GSL2* (Table 3).

**Table 2:** **Important motifs identified in the *GSL1 *** **promoter region**

**Motif number**	**Related function**	**ID/IUPAC**	**Motif sequence**	**Organism described**	**Sequence in DM**	**Position**		**Strand**	**Polymorphism for disrupted motifs**^**a**^
						**Start**	**End**		
						**Start**	**End**		
1	Disease responsive	BIHD1OS	TGTCA	*Oryza sativa*	TGTCA	-1024	-1028	-	CKSV
					TGTCA	-878	-874	+	
					TGTCA	-873	-869	+	
					TGTCA	-385	-389	-	
					TGTCA	-110	-106	+	
2	Pathogen- and salt- induced expression (GT-1 motif)	GT1GMSCAM4	GAAAAA	*Glycine max*	GAAAAA	-1678	-1673	+	
					GAAAAA	-1069	-1064	+	
					GAAAAA	-918	-923	-	
					GAAAAA	-826	-821	+	C^#^
					GAAAAA	-706	-701	+	S^#^V^#^
					GAAAAA	-505	-500	+	C^#^SV
3	Light regulated (I box/I-box)	IBOXCORE	GATAA	Angiosperms	GATAA	-1834	-1838	-	
					GATAA	-1545	-1541	+	CKSV
					GATAA	-1483	-1487	-	S^#^V^#^
					GATAA	-1463	-1459	+	CKSV
					GATAA	-1242	-1238	+	C
					GATAA	-1121	-1117	+	
					GATAA	-1109	-1105	+	
					GATAA	-834	-830	+	
					GATAA	-537	-541	-	
					GATAA	-235	-239	-	CKSV
					GATAA	-117	-121	-	K^#^
4	Dehydration response (MYB recognition site)	MYB1AT	WAACCA	*Arabidopsis thaliana*	AAACCA	-1927	-1922	+	V^#^
					AAACCA	-703	-698	+	S^#^V^#^
					TAACCA	-173	-168	+	
					AAACCA	-15	-10	+	
5	Transcriptional activator (core motif of *MybSt1*, a potato MYB homolog binding site)	MYBST1	GGATA	*Solanum tuberosum*	GGATA	-1556	-1552	+	CKSV
					GGATA	-1299	-1303	-	
					GGATA	-1122	-1118	+	
					GGATA	-835	-831	+	
					GGATA	-116	-120	-	K^#^
6	Rosette leaf- and root-specific	RAV1AAT	CAACA	*Arabidopsis thaliana*	CAACA	-1834	-1830	+	
					CAACA	-1320	-1316	+	
					CAACA	-1242	-1246	-	
					CAACA	-981	-977	+	
					CAACA	-339	-335	+	
					CAACA	-281	-285	-	
					CAACA	-169	-165	+	
7	Axillary bud-specific (sugar-repressive element)	SREATMSD	TTATCC	*Arabidopsis thaliana*	TTATCC	-1117	-1122	-	
					TTATCC	-830	-835	-	
					TTATCC	-121	-116	+	K^#^
8	Root apical meristem-specific	WUSATAg	TTAATGG	*Oryza sativa*	TTAATGG	-15	-21	-	

Analysis used Genomatix-MatInspector [41] based on PLACE [42]. The approximate positions of the motif numbers are indicated on Figure 1A. ^a^All four tetraploid genotypes contained all motifs. However, allelic polymorphisms involving SNPs were occasionally observed in some motifs of these genotypes as indicated by C (1021/1), K (Karaka), S (Summer Delight), and V (VT^n^62-33-3). ^#^indicates an indel in the motif of at least one allele of the indicated genotype.

**Table 3:** **Important motifs identified in the *GSL2 *** **promoter region**

**Motif number**	**Related function**	**ID/IUPAC**	**Motif sequence**	**Organism described**	**Sequence in DM**	**Position**		**Strand**	**Polymorphism for disrupted motifs**^**a**^
						**Start**	**End**		
1	Transcriptional activator (MYB binding site)	MYBPLANT	MACCWAMC	*Antirrhinum majus*	CACCTACC	−353	−346	+	
				*Phaseolus vulgaris*					
				*Petunia hybrida*					
				*Arabidopsis thaliana*					
				*Zea mays*					
				*Petroselinum crispum*					
2	Tissue-specific expression (RY repeat motif)	RYREPEATVFLEB4	CATGCATG	*Phaseolus vulgaris*	CATGCATG	−182	−175	+	
				*Glycine max*					
				*Vicia faba*					
				*Oryza sativa*					
				*Arabidopsis thaliana*					
3	Sucrose responsive element (regulation of a potato tuber storage protein)	SURE1STPAT21	AATAGAAAA	*Solanum tuberosum*	AATAGAAAA	−101	−109	-	
4	Cytokinin responsive	CPBCSPOR	TATTAG	*Cucumis sativus*	TATTAG	−310	−315	-	
5	Pathogen- and salt- responsive (GT-1 motif)	GT1GMSCAM4	GAAAAA	*Glycine max*	GAAAAA	−273	−278	-	CKSV
					GAAAAA	−193	−188	+	
					GAAAAA	−143	−148	-	
					GAAAAA	−105	−110	-	
					GAAAAA	−21	−26	-	CV
6	Gibberellin responsive	MYBGAHV	TAACAAA	*Hordeum vulgare*	TAACAAA	−467	−473	-	V^#^
				*Oryza sativa*					
7	Transcriptional activator (core motif of *MybSt1*, a potato MYB homolog binding site)	MYBST1	GGATA	*Solanum tuberosum*	GGATA	−437	−433	+	
8	Auxin induction & tissue-specific expression	NTBBF1ARROLB	ACTTTA	*Agrobacterium rhizogenes*	ACTTTA	−374	−369	+	
9	Axillary bud-specific (sugar-repressive element)	SREATMSD	TTATCC	*Arabidopsis thaliana*	TTATCC	−432	−437	-	
10	Sugar responsive	WBOXHVISO1	TGACT	*Hordeum vulgare*	TGACT	−13	−17	-	
11	Root apical meristem-specific	WUSATAg	TTAATGG	*Oryza sativa*	TTAATGG	−224	−230	-	

Analysis used Genomatix-MatInspector [41] based on PLACE [42]. The approximate positions of the motif numbers are indicated on Figure 1B. ^a^All four tetraploid genotypes contained all motifs. However, allelic polymorphisms involving SNPs were occasionally observed in some motifs of these genotypes as indicated by C (1021/1), K (Karaka), S (Summer Delight), and V (VT^n^62-33-3). V^#^ indicates an insertion into the motif in at least one allele of VT^n^62-33-3.

Eight different key motifs were identified in the *GSL1* promoter, which are repeated up to eleven times resulting in a total of 42 motifs (Table 2). These involve motifs associated with roles for response to disease and biotic stress, abiotic stresses, light induction, and plant development and were found in the genome sequence of DM as well as all four tetraploid genotypes. However, allelic polymorphisms involving disruptions of these motifs were occasionally observed in the tetraploid genotypes. Polymorphic SNPs were observed in seven of the 42 motifs, with polymorphic indels also observed for nine motifs (Table 2).

In the *GSL2* promoter one key motif was identified that occurs five times and ten other key motifs were identified that occur only once (Table 3). Similar to the promoter of the *GSL1* gene, these have known roles associated with biotic stress, abiotic stress, and development. Additional motifs present in the *GSL2* promoter are associated with sugar signaling and hormone responses. These fifteen motifs were all observed in the four tetraploid genotypes, although two were observed to be polymorphic for SNPs and one was polymorphic for an insertion into at least one allele of the genotype VT^n^62-33-3 (Table 3).

The conservation of these motifs across the genome of DM and all four re-sequenced tetraploid genotypes substantiates their importance. Their presence in the *GSL1* and *GSL2* promoter regions aligns with the transcriptional expression of the respective genes observed by previous northern analysis [2,3] and/or the FPKM data (Figure 2). The presence of allelic polymorphisms involving sequence disruptions in some of these motifs could be representative of alleles with potentially altered transcriptional expression of the *GSL1* and *GSL2* genes.

### Antisense knockdown of GSL expression

Using our standard *Agrobacterium*-mediated transformation protocol for potato [43], we failed to recover any transformants of potato cultivar Iwa with antisense constructs for either the *GSL1* or *GSL2* genes. Over 100 leaf explants were subjected to *Agrobacterium*-mediated transformation in each of three experiments for both *GSL* genes using our well established protocol. We would normally expect to recover at least one regenerated transformant per leaf explant for the potato cultivar Iwa when selecting for the kanamycin resistance marker gene used on the binary vector. This expected frequency was achieved in concurrent related experiments using the *GSL* sense constructs [44]. However, a total of only 33 and 49 putative transformed cell colonies were recovered from all three co-cultivation experiments with *Agrobacterium* containing the *GSL1* and *GSL2* antisense constructs, respectively (Additional file 7: Table S5). All potato cell colonies transformed with the antisense constructs failed to continue growth and eventually senesced and died before complete shoots were regenerated (Additional file 8: Figure S3). The senescing cultures were sub-cultured onto medium without Timentin™ and DNA was extracted from those exhibiting no *Agrobacterium* growth. PCR using primers that bridged the *Lhca3* promoter and the antisense *GSL* coding regions confirmed that these cell colonies were transformed with the intended construct prior to their death (Additional file 9: Figure S4). The same DNA samples failed to amplify PCR products using primers specific to the *Agrobacterium virG* gene. This confirms the absence of *Agrobacterium* cells in the plant tissue that would otherwise compromise the PCR testing of the transformed potato cell colonies.

The lethality of antisense knock-down expression of *GSL1* and *GSL2* suggests an essential role of the *GSL* gene family for potato development. A previous study achieved partial silencing of *GSL1* in potato by expressing an antisense RNA under the control of the 35S promoter. This resulted in plants with reduced height and smaller leaves resulting from reduced cell division, changed leaf metabolism and cell wall composition [32]. The *Lhca*3.St.1 promoter used in the present study is known to confer higher and more stable transgene expression than the 35S promoter [45]. Consequently, the lethality of *GSL1* and *GSL2* antisense knock-down under the control of the *Lhca3* promoter is not unexpected given the dramatic phenotypes observed with the partial silencing from the use of the 35S promoter [32]. It is plausible that these *GSL1* or *GSL2* knock-down impacts, resulting from antisense expression driven by either the 35S or the *Lhca3* promoters, could also arise by interference in expression of other closely related *GSL* and *GASA* genes. The three most closely related genes to *GSL1* show 68-78% identity in exon regions, whereas the identity with all the other related genes was only 42-56% (Additional file 3: Table S1). For *GSL2*, the related *GSL* and *GASA* genes have only 44-60% identity in exon regions (Additional file 3: Table S1). Although this level of identity may be sufficient to trigger a partial knock-down of these related genes, it is unlikely to result in complete knock-down necessary for lethality. Therefore, the lethality of *GSL1* and *GSL2* antisense expression under the control of the *Lhca3* promoter can be attributed to knock-down of the *GSL1* and *GSL2* genes.

## Conclusions

GSL1 and GSL2, Gibberellin Stimulated-Like proteins (also known as Snakin-1 and Snakin-2), are cysteine-rich peptides from potato (*Solanum tuberosum* L.) with antimicrobial properties [2,3]. Given their *in vitro* antimicrobial activity, the *GSL1* and *GSL2* genes are often considered to play important roles in the innate defence against invading microorganisms [2,3,6] and/or to be a key determinant during the interaction between plants and pathogens [25,26]. In other species similar GSL/GASA proteins are hypothesised to play diverse biological roles in several aspects of plant development, plant responses to biotic or abiotic stress through their participation in hormone crosstalk, and redox homeostasis [31]. To further the understanding of the biological roles of GSL proteins, we undertook a thorough analysis of the structure and expression of these genes in potato.

We isolated and sequenced the coding regions and cDNAs for both *GSL1* and *GSL2* genes from the potato cultivar Iwa. This revealed two alleles (a1 and a2) for the *GSL1* gene (GenBank accessions FJ195646 and FJ195647) and two alleles (b1 and b2) for the *GSL2* gene (GenBank accessions EU848497 and EU848498). Alignment of the genomic and cDNA sequences confirmed the exon and intron regions in both the *GSL1* and *GSL2* genes (Figure 1). The *GSL1* gene consists of two exons of 82 and 187 nucleotides interrupted by a single intron of 525 nucleotides. The *GSL2* gene is composed of three exons of 87, 46 and 182 nucleotides respectively, alternating with two introns of 268 and 172 nucleotides.

We have also characterised the full length genes for both *GSL1* (chromosome 4) and *GSL2* (chromosome 1) using the genome sequence of diploid potato [33], coupled with further next generation sequencing of four highly heterozygous tetraploid potato genotypes; cultivars Summer Delight and Karaka, and breeding lines 1021/1 and VT^n^62-33-3. The frequency of SNPs in *GSL1* and *GSL2* was very low with only one SNP every 67 and 53 nucleotides in exon regions of *GSL1* and *GSL2*, respectively (Table 1, Additional file 4: Table S2), similar to other highly conserved housekeeping genes in potato (Additional file 4: Table S2).

Specific promoter motifs were also highly conserved among multiple alleles representing the 17 haplotypes from DM and the four re-sequenced tetraploid genotypes (Tables 2 and 3), suggesting their importance for biological function. Analysis of comprehensive RNA-seq data substantiated the role of specific promoter motifs in transcriptional control of gene expression (Figure 2). FPKM analysis established that *GSL2* was expressed at a higher level than *GSL1* in 30 out of 32 libraries, often by an order of magnitude. Furthermore, both the *GSL1* and *GSL2* genes exhibited constitutive expression that was not up-regulated in response to biotic or abiotic stresses, hormone treatments or wounding. The FPKM analysis did not always agree with previous northern analysis [2,3], although closely matched conclusions from the analysis of *Arabidopsis thaliana* plants transgenic for GUS fusions to the potato *GSL1* promoter [40].

The *GSL1* and *GSL2* genes from potato are very highly conserved suggesting they contribute to an important biological function. The known antimicrobial activity of the GSL proteins, coupled with the FPKM analysis from RNA-seq data, suggests that both genes contribute to the constitutive defence barriers in potatoes. The lethality of antisense knock-down expression of *GSL1* and *GSL2*, coupled with the rare incidence of SNPs in these genes, suggests an essential role for this gene family. These features are consistent with the GSL protein family playing a role in several aspects of plant development and plant defence responses.

## Methods

### Extraction of potato DNA and RNA for analysis of *GSL* genes

For cloning and sequencing of the *GSL* genes, genomic DNA was isolated from *in vitro* shoots of potato, *Solanum tuberosum* L., cv Iwa based on the method described by Bernatzky and Tanksley [46]. Total RNA was isolated from the youngest, fully expanded leaves of 2 month old greenhouse-grown Iwa potato plants using the Illustra RNAspin Mini Isolation Kit (GE healthcare, Buckinghamshire, UK), including DNase treatment according to the manufacturer’s instructions. The integrity of the total RNA was checked by electrophoresis in 1% agarose gel in Tris-acetate-EDTA (TAE) buffer and quantity was determined with a NanoVue™ Spectrophotometer (GE healthcare).

### PCR isolation of *GSL* genes

Primers GSL1-F2 (5’-AAATGAAGTTATTTCTATTAACTCTGC-3’) and GSL1-R2 (5’-TGTGAAGACGCAAATATAACCAC-3’) were designed based on the reference gene sequence of *StGSL1* (Genbank accession AJ320185) to isolate the *GSL1* gene. The reference gene sequence of *StGSL2* gene (Genbank accession AJ312424) was used to design the primers GSL2-F (5’-AAATATTTCAAATTCCAATGGC-3’) and GSL2-R (5’-CAATACAATGCAAACCAGAACAA-3’) to isolate the *GSL2* gene. PCRs were carried out in a Mastercycler (Eppendorf, Hamburg, Germany). The 50 μl PCR mix contained 1x Expand High Fidelity^PLUS^ Reaction Buffer containing 1.5 mM MgCl_2_, 0.2 mM of each dNTP, 0.4 μM of each primer, 1 μl of DNA (~100 ng) and 2.5 U of Expand High Fidelity *Taq* DNA polymerase (Roche Applied Science, Mannheim, Germany). The conditions for PCR for the *GSL1* gene were: 93°C for 1 min, 35 cycles of 30 s 92°C, 30 s 57°C, 90 s 72°C, followed by 6 min extension at 72°C. For the *GSL2* gene, PCR was performed using the same PCR conditions with 58°C annealing temperature. Amplified products were separated by electrophoresis in a 1% agarose gel in 1xTAE buffer and visualized under UV light after staining with ethidium bromide. Additional primers were designed to flank the previously designed primer regions and following PCR the products were sequenced to confirm the authenticity of the sequence over the previous primer regions.

### Cloning and sequencing of *GSL* genes

PCR fragments of the expected size (813 bp for *GSL1* and 953 bp for *GSL2*) were extracted from an agarose gel using a QIAquick gel extraction kit (QIAGEN, Hilden, Germany) and cloned into pGEM®-T Easy vector (Promega, Mannheim, Germany). The resulting plasmids were transformed into Subcloning Efficiency™ DH5α™ Competent Cells (Invitrogen, Carlsbad, CA, USA) according to manufacturer’s instructions. Plasmid DNA from white clones was isolated using High Pure Plasmid Isolation Kit (Roche Applied Science) and tested by restriction analysis using FastDigest® *Not*I enzyme (Fermentas, Hanover, Maryland, USA) to identify whether they contained *GSL* gene inserts. Plasmid DNA from 16 clones of each *GSL* gene was sequenced using Applied Biosystems BigDye® Terminator v3.1 kit. Sequencing reactions were analysed using an ABI 3130xl automated sequencer (Applied Biosystems, Foster City, USA). Each fragment was sequenced from both directions individually using 3.2 pmole of primer. M13 forward and M13 reverse primers were used individually as sequencing primer in each sequencing reaction. Vector NTI Advance 10 software package (Invitrogen) was used to analyse the sequences and assemble into contigs.

### Sequencing the coding regions of *GSL* genes

First-strand cDNA was synthesised from isolated RNA using the SuperScript® VILO™ cDNA Synthesis Kit (Invitrogen, Carlsbad, USA) according to the manufacturer’s instructions. VILO™ Reaction Mix contains random primers, MgCl_2_ and dNTPs in a buffer formulation. Single-stranded cDNA was then used as a template in the PCR reactions using the Expand High Fidelity^PLUS^ PCR system (Roche Applied Science). Approximately 50 ng of cDNA, corresponding to the amount of total RNA isolated from Iwa plants, was used as a template. The PCRs were carried out in a C1000™ Thermal Cycler (Bio-Rad). The 50 μl PCR mix contained 1x Expand High Fidelity^PLUS^ Reaction Buffer containing 1.5 mM MgCl_2_, 0.2 mM of each dNTP, 0.4 μM of each primer and, 2.5 U of Expand High Fidelity *Taq* DNA polymerase (Roche Applied Science). For the *GSL1* coding region, the primers were GSL1-exonF2 (5’-ATGAAGTTATTTCTATTAACTCTGCTTT-3’) and GSL1-exonR2 (5’-TCAAGGGCATTTAGACTTGC-3’). For the *GSL2* coding region, the nucleotide sequences of the primers were GSL2-exonF (5’-ATGGCCATTTCGAAAGC-3’) and GSL2-exonR (5’-TTAAGGGCATTTACGTTTGTT-3’).

The PCR conditions for the *GSL1* coding region were: 94°C for 2 min, 34 cycles of 30 s 94°C, 30 s 59°C, 30 s 72°C, followed by 7 min extension at 72°C. The PCR for the *GSL2* coding region was performed using the same PCR conditions with 57°C annealing temperature. PCR products were separated by electrophoresis in a 2% agarose gel in TAE buffer and visualized under UV light after staining with ethidium bromide.

PCR products were purified using the Illustra GFX™ PCR DNA and Gel Band Purification Kit (GE Healthcare) according to the manufacturer’s recommendations and sequenced directly using Applied Biosystems BigDye® Terminator v3.1 kit. Sequencing reactions were analysed using an ABI 3130xl Genetic Analyzer (Applied Biosystems, Foster City, USA). Each fragment was sequenced from both directions individually using 3.2 pmole of primer. The primers described above were used individually as sequencing primer in each sequencing reaction. Sequences of *GSL1* and *GSL2* coding regions were analysed and aligned with their reference sequences by MUSCLE alignment method [47] using Geneious software [38].

### Next generation sequencing

Genomic DNA was isolated from young shoots of greenhouse grown potato plants of cultivars Summer Delight and Karaka, and breeding lines 1021/1 and VT^n^62-33-3, based on the method described by Bernatzky and Tanksley [46]. Illumina short insert read pair data were generated for each line using the Illumina GAIIX platform (Illumina, San Diego).

### Transcript profiling

Transcriptome analyses were performed using data sets produced previously by The Potato Genome Sequencing Consortium [33]. Transcriptome sequences were generated from 32 libraries of genotype DM using RNA-seq with the Illumina Genome Analyser II platform. These 32 libraries represent a wide range of tissues/organs as well as abiotic, biotic and plant growth regulator treatments. Full experimental details are described in the Supplementary Material and Table S4 of The Potato Genome Sequencing Consortium [33]. The number of expressed genes and RNA-seq reads for each of the libraries is presented in Massa et al. [48]. To provide a normalized unit to allow comparisons within and between samples, the abundance of transcripts was expressed in fragments per kilobase of exon model per million mapped reads (FPKM) as implemented in Cufflinks [49].

### Bioinformatic analysis

Illumina short insert paired-end reads of the four tetraploid potato lines were aligned to the reference genome using BWA [36]. For 1021/1, VT^n^62-33-3, Karaka and Summer Delight, 60.6 × 10^6^ (71%), 53.4 × 10^6^ (69%), 49.4 × 10^6^ (72%) and 37.1 × 10^6^ (72%) read pairs were mapped to the reference genome, respectively. This resulted in approximately 9 to 15 fold coverage of the ~840 MB potato genome for these four genotypes. Alignments were further analysed using SAMtools [37]. Single nucleotide polymorphisms were detected using an in-house tool, polySNP (https://github.com/mfiers/polysnp). PolySNP calls SNPs based on Samtools mpileup mapping quality scores. Only high confidence SNPs for uniquely mapped reads with sequence scores above phred 15 were considered. SNPs had to be present in at least three reads to be counted. Output from polySNP was validated by manual confirmation using the software package Geneious [38].

The analysis of promoter regions for motifs predicted to be involved in transcription factor binding sites was performed using the Genomatix MatInspector software [41] with the selection of Plant IUPAC Library based on PLACE [42].

### Construction of the expression cassette

Sequence information derived from the *StLhca3* promoter and terminator regions was used to design a potato expression cassette into which coding regions of other potato genes can be cloned. A region consisting of nucleotides 1–600 from the *StLhca3* promoter (GenBank accession EU234502) and a region consisting of nucleotides 101–487 from the *StLhca3* terminator (GenBank accession EU293853) were adjoined *in silico* to generate a unique *Psi*I restriction site at their junction. These sequences were synthesized as a single 988 bp fragment (Genscript Corporation, Piscataway, NJ, USA) and cloned into pUC57 to produce pStLhca3cas (Additional file 10: Figure S5A).

### Construction of antisense vectors

PCR was performed to isolate blunt-end *GSL1* and *GSL2* sequences from the pGEM®-T Easy plasmids harbouring specific GSL alleles described above. Vent^R^ DNA polymerase (New England BioLabs, Massachusetts, USA) was used to isolate blunt-end fragments from the a1 allele of *GSL1* (GenBank accession FJ195646) using the primers GSL1-F2 and GSL1-R2 and the b1 allele of *GSL2* (GenBank accession EU848497) using the primers GSL2-F and GSL2-R. PCR products of the expected size (813 bp for the a1 allele of *GSL1* gene and 955 bp for the b1 allele of *GSL2* gene) were gel-purified using QIAquick Gel Extraction Kit (QIAGEN). Quick Blunting Kit (New England BioLabs) was used for phosphorylation of the 5’ ends of the blunt-ended DNA fragments. The fragments were ligated into the *Psi*I site of the expression cassette (pStLhca3cas) using T4 DNA Ligase (New England BioLabs). One Shot® TOP10 Electrocomp™ *E. coli* Cells (Invitrogen) were transformed with DNA from blunt-end ligation reactions. Plasmid DNA from clones was isolated using High Pure Plasmid Isolation Kit (Roche Applied Science) and tested by restriction analysis using *Hin*dIII enzyme. The recombinant plasmid was sequenced using Applied Biosystems BigDye® Terminator v3.1 kit to confirm the orientation of the expression cassette. The primers Cab-Fa (5’-TTCTAGTGGAGCTAAGTGTTCA-3’) and Cab-Ra (5’-TGTTACATTACACATAAGAGAAGG-3’) were used individually as sequencing primers in each sequencing reaction. Sequencing reactions were analysed using an ABI 3130xl Genetic Analyzer (Applied Biosystems, Foster City, USA). Plasmids containing *StLhca3* expression cassettes with the *GSL* genes in the sense orientation and those with inserts in the opposite orientation (antisense expression cassettes) were identified by sequencing.

Plasmids of antisense expression cassettes were digested with *Hin*dIII and the resulting fragments, *Lhca3-StGSL1* (1631 bp) and *Lhca3-StGSL2* (1770 bp) were blunt-ended using Quick Blunting Kit (New England BioLabs) and ligated individually into the blunt-ended *Not*I site of the binary vector pMOA33 [50] using T4 DNA Ligase (New England BioLabs). Ligation products were transformed into MAX Efficiency® DH5α^TM^ Competent Cells (Invitrogen). Colonies were screened using colony PCR with Cab-Fa and Cab-Ra primers to identify intact clones. Individual colonies were picked using a sterile pipette tip and resuspended in 10 μl of PCR mix. Each 10 μl PCR mix contained 1xThermoPol Reaction Buffer, 0.2 mM of each dNTP, 0.2 μM of each primer and, 0.4 U of *Taq* DNA polymerase (New England BioLabs). The PCRs were carried out in a Mastercycler (Eppendorf). The conditions for PCR were: 94°C for 4 min, 34 cycles of 15 s 93°C, 30 s 55°C, 90 s 72°C followed by 10 min extension at 72°C.

Plasmid DNA from clones selected by colony PCR was isolated using the QIAprep Spin Miniprep kit (QIAGEN). The orientation of the expression cassettes within the T-DNA in the binary vectors, pMOA33-Lhca3-antiGSL1 and pMOA33-Lhca3-antiGSL2, was tested by restriction analysis (*Eco*RV and *Sca*I for pMOA33-Lhca3-antiGSL1; *Eco*RV and *Xho*I for pMOA33-Lhca3-antiGSL2) to select a binary vector that contains the *Lhca3* promoter adjacent to the right border within the T-DNA.

### Potato transformation

The resulting binary vectors (Additional file 10: Figures S5B and S5C) were transferred to the disarmed *Agrobacterium tumefaciens* strain EHA105 [51] using the freeze-thaw method [52]. *Agrobacterium* cultures harbouring the binary vectors were cultured overnight on a shaking table at 28°C in LB broth supplemented with 300 mg l^-1^ spectinomycin. Leaf segments from virus-free plants of potato (cultivar Iwa) were transformed using our well established protocol [43] with 100 mg l^-1^ kanamycin to select for transformed potato cells and 200 mg l^-1^ Timentin™ to prevent *Agrobacterium* overgrowth.

### Molecular confirmation of transformation

Independently-derived putative transformed potato cell colonies were sub-cultured onto culture medium without Timentin™. Genomic DNA was extracted from those exhibiting no *Agrobacterium* growth using a modified CTAB method [53]. To confirm the presence of the antisense-GSL constructs in the cell colonies, primers specific to the *Lhca3* promoter region and the *GSL* genes were used to avoid endogenous gene amplification. The PCR for the *Lhca3-antiGSL1* gene used the primers Cab-Fa (5’-TTCTAGTGGAGCTAAGTGTTCA-3’) and GSL1-F1 (5’-ACCCTTCTCTCATTCAAACT-3’) with a predicted amplicon of 840 bp. The PCR for the *Lhca3-antiGSL2* gene used the primers Cab-Fa and GSL2-bF1 (5’-TCAGACCGATCAAGTGGTGA-3’) with a predicted amplicon of 940 bp. The following PCR conditions were used: 1 cycle at 94°C for 1 min, 34 cycles of 20 s 93°C, 20 s 55°C, 80 s 72°C, followed by a 3 min extension at 72°C. Finally, primers specific to the *Agrobacterium virG* gene, GMT24virGF (5’-GCGGTAGCCGACAG-3’) and GMT25virGR (5’-GCGTCAAAGAAATA-3’) producing a predicted amplicon of 692 bp were used to investigate the possible presence of *Agrobacterium* contamination remaining in the plant tissue. The PCR conditions were 2 min at 94°C, then 34 cycles of 30 s 94°C, 30 s 45°C, 30 s 72°C, followed by a 5 min extension at 72°C. All PCRs were conducted in 10 μL reactions containing 1x ThermoPol Reaction Buffer [20 mM Tris–HCl, 10 mM KCl, 10 mM (NH_4_)_2_SO_4_, 2 mM MgSO_4_, 0.1% Triton X-100, pH 8.8 at 25°C], 0.2 mM of each dNTP, 0.2 μM of each primer and 0.4 U of *Taq* DNA Polymerase (New England Biolabs). PCRs were carried out in a Mastercycler (Eppendorf, Hamburg, Germany) and amplified products were separated by electrophoresis in a 1% agarose gel in 1xTAE buffer at 5.5 V/cm for 40 min and visualized under UV light after staining with ethidium bromide (5 mg l^-1^) for 15 min.

## Competing interests

The authors declare that they have no competing interests.

## Authors’ contributions

SMe cloned and sequenced cDNA and genomic DNA for the *GSL* genes, constructed the antisense vectors, annotated the sequences, and analysed promoter motifs. SJT and MWEJF performed bioinformatic analyses and determined SNP frequencies. MWEJF designed the polySNP tool. PJB interpreted the expression profile data of *GSL* genes and assisted in sequence annotation. JML, SMo and EEJ contributed to the cDNA sequencing, annotation of *GSL* genes, and carried out the potato transformations. AJC conceived the study, coordinated data analysis and wrote the manuscript. JMEJ conceived the study, undertook the next generation sequencing and data generation, and wrote the manuscript. All authors contributed to, read and approved the final manuscript.

## Supplementary Material

Additional file 1: Figure S1 Nucleotide sequence of the *GSL1* gene with 5’upstream regulatory and terminator regions from potato DM; derived from The Potato Genome Sequencing Consortium [33]. Numbering is defined by the putative transcription start site (TSS, +1) predicted at 33 nt from the first base of the translation start site (ATG), based on a plant dimer motif YR Rule (TG, -1/+1). Putative *cis*-elements TATA-box (−32 to −27, highlighted violet), a pyrimidine patch (Y Patch, -26 to −20, highlighted pink) and CAAT-box (−48 to −44, highlighted red) were also identified. Other nucleotide sequences highlighted are: positions of promoter motifs annotated as numbered ovals on Figure 1A and listed in Table 2 (blue); 5’UTR (grey); exons (yellow); and intron (green). The start and stop codons are marked in red font. Click here for file

Additional file 2: Figure S2 Nucleotide sequence of the *GSL2* gene with 5’upstream regulatory and terminator regions from potato DM; derived from The Potato Genome Sequencing Consortium [33]. Numbering is defined by the putative transcription start site (TSS, +1) predicted at 38 nt from the first base of the translation start site (ATG), based on a plant dimer motif YR Rule (TG, -1/+1). Putative *cis*-elements TATA-box (−50 to −45, highlighted violet), a pyrimidine patch (Y Patch, -59 to −51, highlighted pink) and hypothetical CAAT-box (−65 to −61, highlighted red) were also identified. Other nucleotide sequences highlighted are: positions of promoter motifs annotated as numbered ovals in Figure 1B and listed in Table 3 (blue); 5’UTR (grey); exons (yellow); and introns (green). The start and stop codons are marked in red font. Click here for file

Additional file 3: Table S1 List of *GSL* and *GASA* genes and their genetic position in potato. The chromosomal location is supported by super-scaffolds anchored via a genetic map generated for DM [54], incorporating information from RH and tomato, or a genetic map of RH generated by whole genome profiling (WGP) [55]. Locations of the *GSL1* and *GSL2* genes were identified by the superscaffold location on the physical map given in the agp file generated by the Potato Genome Sequencing Consortium (PGSC) (http://solanaceae.plantbiology.msu.edu/pgsc_download.shtml). The alignment of coding regions to determine identity to *GSL1* and *GSL2* used MUSCLE [47] and was based on allele a1 for *GSL1* (FJ195646) and allele b1 for *GSL2* (EU848498). Click here for file

Additional file 4: Table S2 SNP frequency in all GSL and GASA-like genes and a number of housekeeping genes in potato. The next generation sequence data from four tetraploid potato genotypes (‘Karaka’, ‘Summer Delight’, 1021/1, VT^n^62-33-3), plus the diploid RH [33], were aligned with the genome of DM [33]. Output is from polySNP tool with stringent calling (https://github.com/mfiers/polysnp). SNP frequency is given as nucleotides/SNP; ‘-‘ indicates no SNPs present. Click here for file

Additional file 5: Table S3 Motifs identified in the DM *GSL1* promoter. Analysis used Genomatix-MatInspector [41] based on PLACE [42]. Click here for file

Additional file 6: Table S4 Motifs identified in the DM *GSL2* promoter. Analysis used Genomatix-MatInspector [41] based on PLACE [42]. Click here for file

Additional file 7: Table S5 Transformation of potato antisense constructs of the *GSL1* and *GSL2* genes. Results are presented for three independent *Agrobacterium*-mediated transformation experiments of potato cultivar Iwa using the binary vectors pMOA33-Lhca3-antiGSL1 (Additional file 10: Figure S4B) and pMOA33-Lhca3-antiGSL2 (Additional file 10: Figure S4C). Click here for file

Additional file 8: Figure S3 Senescing potato cell colonies transformed with antisense constructs of the *GSL1* gene. Identical results were obtained for the antisense construct of the *GSL2* gene. Click here for file

Additional file 9: Figure S4 PCR confirmation of transgenic status of potato cell colonies transformed with antisense constructs of the *GSL1* and *GSL2* genes. Click here for file

Additional file 10: Figure S5 Plasmids constructed and used in this study. A. pStLhca3cas; B. pMOA33-Lhca3-antiGSL1; C. pMOA33-Lhca3-antiGSL2. Click here for file
