# Supplementary material for: Structure and expression of GSL1 and GSL2 genes encoding gibberellin stimulated-like proteins in diploid and highly heterozygous tetraploid potato reveals their highly conserved and essential status
Source: BMC Genomics. 2014 Jan 2;15:2. doi: 10.1186/1471-2164-15-2 (PMC3890649; doi:10.1186/1471-2164-15-2)
Supplement: Additional file 1: Figure S1 — Nucleotide sequence of the GSL1 gene with 5’upstream regulatory and terminator regions from potato DM; derived from The Potato Genome Sequencing Consortium [33]. Numbering is defined by the putative transcription start site (TSS, +1) predicted at 33 nt from the first base of the translation start site (ATG), based on a plant dimer motif YR Rule (TG, -1/+1). Putative cis-elements TATA-box (−32 to −27, highlighted violet), a pyrimidine patch (Y Patch, -26 to −20, highlighted pink) and CAAT-box (−48 to −44, highlighted red) were also identified. Other nucleotide sequences highlighted are: positions of promoter motifs annotated as numbered ovals on Figure 1A and listed in Table 2 (blue); 5’UTR (grey); exons (yellow); and intron (green). The start and stop codons are marked in red font. [file 1471-2164-15-2-S1.pdf]

Supplementary Figure 1. Nucleotide sequence of the *GSL1* gene with 5'upstream regulatory and terminator regions from potato DM; derived from The Potato Genome Sequencing Consortium [33]. Numbering is defined by the putative transcription start site (TSS, +1) predicted at 33 nt from the first base of the translation start site (ATG), based on a plant dimer motif YR Rule (TG, -1/+1). Putative *cis*-elements TATA-box (-32 to -27, highlighted violet), a pyrimidine patch (Y Patch, -26 to -20, highlighted pink) and CAAT-box (-48 to -44, highlighted red) were also identified. Other nucleotide sequences highlighted are: positions of promoter motifs annotated as numbered ovals on Figure 1A and listed in Table 2 (blue); 5'UTR (grey); exons (yellow); and intron (green). The start and stop codons are marked in red font.

-1967 AAAAGCATTAAAACATCATAGGACAAAAATCTGCCCATGCAAACCACACATATGTCCCATGCTAGAACAAAACATGATGG

-1887 TGGGGGAAAGAAAATGAAAACGAAAATGAAAAGCACTATTACACTTAAATTATCAACAAACGACAAGTCATATCATATCA

-1807 TAATTATAATAAGTGGTAGTAACATGCATCGGCCCCAAATCTTTGCAAAAGATCGTTAATAGTACTCACATCGAATCACT

-1727 CTACTTGTCGTTCCTATTTAGTGCATGAATCACGATAGTGATTATATTAGAAAAATCAAAATATAATTAACTCAGTGCAT

-1647 TGATTTCATTTTATTTTTAGTAATAACTAATTTTGAAAATAGTAGACATTGACTAATTAGTATATGTGAAGATCCGTAAA

-1567 ATTTTAATCATGGATATTCGACGATAAATTTTTAAAATCTATTAGTACTAAGTACTAACTTAGAGCAGAAATTTTCATAG

-1487 TTATCGACCTGTTACATAAGCAGAGATAAATGTGAAATATTATTATACCCCTATTGTATTTATATAAGAACGCAAAGTTA

-1407 CAATAAATACTTTTTTTTTATTTTCCGCTCTCAGTGTTCGGAGCTGATTGAGCACTGCAGGATCTATCTGGGGGGGAGTG

-1327 ACGCTTCCAACAATATTTTCTCCATATCCAAAACTCGATTTTGAGACATTTGATTAAGAATGGTCCATGCACCACAATTC

-1247 ATGTTGATAAAGTTATGATACATATAAAAGATCAATTTAAGAGATTTCAAATGAATTTACAATTTTGTAGCCACTCAATT

-1167 ATTATTGTACGACTTTGGGTTGAATGCTCAAGTGGACTGAAATTTGGATAAATATCTTGATAAAATTAACAAAAGAGAAT

-1087 ACTTGAAAGTTAAAAATTGAAAAAAATAATCTAGAAAGAATAATTCGAACTTCCAAATTTGACATCGATCGTACAAATTT

-1007 GTGAAGAAAACTTAGCGGTTTCGGTACAACACTTTACACAGAAGTGGAGACTTGGAGTGAAGTTAGGTAAAAATATTGCT

-927 TAATTTTTTCATAGAGAACTTTAGGAATTATATTTATTACTATAATTTATGTCATGTCACCTCACCAAAACAGTTCCATA

-847 TTTCTTTTCTTTGGATAAAAGGAAAAAAATTTGTCTAATACGTATTAAAAATCAATCAAAATCTTAATGATCTATGTAGC

-767 CATAAAATTATTTACTTAATTATAAAATTGATGTTTGGCTATAAAAATTCAAACTAAATTTGAAAAACCATTTGAAACTG

-687 TTTTCCAACTCATTTTAACTTTTGAATATATTCATACTCAAATACAAAAAATATAATTAAATAAAATTTTCCGTTCAACT

-607 GTAACTTTATAAATTTCAAATAACAATAATAACTACAAATAAAGTTTGAAAAGAATAAATATAAGCTTATCTTACTCGTA

-527 AAAGCCTATTTTACGAAAGATAGAAAAATGTAACTCTATACTTTCAAATATAAAACAAAAATATTTAAAAATTTATGACG

-447 AAACGTCTGCTTAATAGCCAGCTTGCACAAATGCCAAGCATAGTACAAAAACAGAAGATGACAAAATCATGAATTTTAGC

-367 GTAACCCACTTTTAATGTGGAAATACTCCAACAACCCCACCCCATCCGTATTTATTCGCATTCGATATTTATGAAATTTT

-287 CATGTTGAATGACTTGTTCATATTTAATATTTAAGCCAAAATTATTAATTATCTATATTTAGTGATAGATTAAAATGCGA

-207 ATATATATATATATATTTATTTATTTATTATGATTAACCAACATTATGTATCATGTATTTATTTGAAATGGTATAAATGA

-127 ATTTTTTTATCCTGTTTTGTCAGACCCATCCACAGTTTACTTACTTAATTAGTTATAAAAAACATTTATAGTGGACCACC

-47 AACTTTTCTCCTGGCTATATATTCCCCCATTAAAACCATTTAGTAATGCTTCACTTCATCAAATTTTCAGCTTAGAAAAA

+34 **ATG**AAGTTATTTCTATTAACTCTGCTTTTGGTCACTCTTGTCATTACCCCTTCTCTCATTCAAACTACTATGGCTGGTTC

+114 AAGTAAGTACCAATTCTTCCATCATCTTTAAAATTTGCTACTAGTCTTCTGGAAACATTCACGAGGTAGTAGTAAGGTTT

+194 TCGTATACTCTGCTTTTTTCATACCTTACCAAGTGAAATTTGAATATGTACTTGTTCTCGGGGCATAGCTATAGCCACTC

+274 CAGGGTATCCAATTATACGTCAAATTCTACATTTAAAGAATATATATTTAAAATTGAATACACTTGACAAAAGTTATGCC

+354 TTTAGCTAGTGGTAATGAGTTTTCATTTGAATGAAAGAACTTTGGGTTTGAACCCCAAATAGAACAATATTTTTTTGTTT

+434 TATTTTGACAGCCACACACCATTATTTTTAGACACTTTTAATAAAATTTCTGGCTTAACCACTGATTGTTCTAATATTTT

+514 CACTACTATCCACTAACACAACACATATATAAGATAATTTTGTCCAATAATTTTTTTTTTAAAAGTTATATGTTCTAATT

+594 ATGAGTTTTTTTTTTTTTTTTTAAAGATTTTTGTGATTCAAAGTGCAAGCTGAGATGTTCAAAGGCAGGACTTGCAGACA

+674 GATGCTTAAAGTACTGTGGAATTTGTTGTGAAGAATGCAAATGTGTGCCTTCTGGAACTTATGGTAACAAACATGAATGT

+754 CCTTGTTATAGGGACAAGAAGAACTCTAAGGGCAAATCTAAATGCCCT**TGA**ATTTGATGTATTCCAAATATCTAAGTGGT

+834 TAAATTTGGTCTTCACATATTAAGTTTTCTAGGTATGTTAAAGTTATACATATTGAAAACTAGAAAAAAAAAAAGAAGCTT

+914 TTGTTATTAATAAATAAATGGGTTCTTTTTTGAACTCTTTGGATAGTTTTCTGCTTTGTTTTAAATGTAATATGATTTTG

+994 AATTCTTATAAATAATAATTTAATGTTTGGTTAAGTAGAACAGCTCATATCTCCAACTGCACTTTATGAAATTAGGAATT

+1074 AGTTAGCTTATTTACCCAAATAATCTTTGATTTTTTCTCTTTTTGGGCAAAGATCTAAACTAAATATCAGGGGTGATTCA

+1154 ATAAAATTAATGGGTTAAAATTCATTTTTATTAAGAGACTGGTATTTTTTGAGATTTTTTTTTTTTTTAAAATGTTATACT

+1234 ATTTTTATTTTTAGAAAAAATAAGATCATAAAATTTACTATTAATTAAAAATAAAAAATGAGTAAAAATATCTTTTAACT

+1314 TATGAAAAATGATTCACCAATACCCTCTATTTATCTGTTGGCCTAGTATTATTTATTAACTTGTATTTTTGGCTAAGAAA

+1394 CATCCTCAAACAAACACGTTGAACTTACTTATTATTATTATTGGTCACATATAATAATAAAGGTGTATTGTTTTTATTTT

+1474 TGATAAAGGGAAAAGCTCAAATATGTCATCGAACTTTCAGAAAAGGCTCATTTATGCCATCCGTTAAAAGTTTGGCTCAT

+1554 CTATGCCATTATCGTTTAAGAAAAGGCTCATTCATGCCATTATTTTTAACGGTGGTTTTGCAAAACCATTTTTTACACGT

+1634 GGCCAATTATAATTCGGCCACGTCATTATTTTTTGGGATAAAAATCAAAAATCTGAACAAACATTTTAACGGTGGTTTTG

+1714 CAAAACCATTTTTTACACGTGGCCAATTATAATTCGGCCACGTCATTATTTTTTGGAATAAAAATCAAAATTCTGAACAA

+1794 ACATTTTAACG
