# Supplementary material for: Structure and expression of GSL1 and GSL2 genes encoding gibberellin stimulated-like proteins in diploid and highly heterozygous tetraploid potato reveals their highly conserved and essential status
Source: BMC Genomics. 2014 Jan 2;15:2. doi: 10.1186/1471-2164-15-2 (PMC3890649; doi:10.1186/1471-2164-15-2)
Supplement: Additional file 2: Figure S2 — Nucleotide sequence of the GSL2 gene with 5’upstream regulatory and terminator regions from potato DM; derived from The Potato Genome Sequencing Consortium [33]. Numbering is defined by the putative transcription start site (TSS, +1) predicted at 38 nt from the first base of the translation start site (ATG), based on a plant dimer motif YR Rule (TG, -1/+1). Putative cis-elements TATA-box (−50 to −45, highlighted violet), a pyrimidine patch (Y Patch, -59 to −51, highlighted pink) and hypothetical CAAT-box (−65 to −61, highlighted red) were also identified. Other nucleotide sequences highlighted are: positions of promoter motifs annotated as numbered ovals in Figure 1B and listed in Table 3 (blue); 5’UTR (grey); exons (yellow); and introns (green). The start and stop codons are marked in red font. [file 1471-2164-15-2-S2.pdf]

Supplementary Figure 2. Nucleotide sequence of the *GSL2* gene with 5'upstream regulatory and terminator regions from potato DM; derived from The Potato Genome Sequencing Consortium [33]. Numbering is defined by the putative transcription start site (TSS, +1) predicted at 38 nt from the first base of the translation start site (ATG), based on a plant dimer motif YR Rule (TG, -1/+1). Putative *cis*-elements TATA-box (-50 to -45, highlighted violet), a pyrimidine patch (Y Patch, -59 to -51, highlighted pink) and hypothetical CAAT-box (-65 to -61, highlighted red) were also identified. Other nucleotide sequences highlighted are: positions of promoter motifs annotated as numbered ovals in Figure 1B and listed in Table 3 (blue); 5'UTR (grey); exons (yellow); and introns (green). The start and stop codons are marked in red font.

-616 ATATATAAATTTTATTTTAAAAAAAATTATATCTAAATTCATGATCAAAATTTAATAATTTTACTCTTAAAATTTTAAAT

-536 AATATCAGGTAAATTGATACGAAAGAGTGTTAAAAGAGATATGCATGAATGGTACTTGAATTATTTGTTAGATGGAATCT

-456 TAAGTTCTCTTTTGGCCAAGGATAATATATATAATTAGGAACCAACTTGTTTTAATTAATTACATGTAATGTAGCTTTGT

-376 AGACTTTATGTGTTAGCTATGAACACCTACCAAAGCAATTATATACACTTTGGAAAATGTACTAATAATTCCAGACTCTA

-296 TGATTAAGCTGTTAGATATTTTTCTAATTAAATAATTAAATTCCTTCTCTATTTTAGTAGTATACCCCATTAACCTACTA

-216 CAGTATAGTTAAGCATCACGTACGAAAAAAATCGCATGCATGAAATGAAACCTACTTGTATATATTATTTTTTCTTTTAT

-136 AAAAAAATATTCTTTTTGCTTTTGTGTTTTTCTATTTTATTTGTCTTCATATACATGAACCTTATATACTTCCACTATTT

-56 CCCCCCTATAAATACCCCATTTACTAATCATTTTTCATAAGTCACACCAAAAGATTGAAGCTTATCAATTTATAGAAAAA

+25 TATTTCAAATTCCA**ATG**GCCATTTCGAAAGCTCTCTTTGCTTCATTACTTCTCTCCTTGCTCCTTCTCGAGCAAGTCCAA

+105 TCTATTCAGACCGATCAAGTGGTGAGTTATTTTATTTGTTCCTTAGCAATTTACTTAAAAATTATATCTATGCTACGTAT

+185 ATTTCTTCGGTTCTAAAATAGAAGAACTCATGATAAATAGTCTAAGAGGTTTGAATTACCGTTTCATCATGAATTTATGT

+265 GGAAAAAAAAAATCTAGTTGACTGTCTAAATATATAGTTAAGAATTTAATCATACTCCTTAATTTAAAGTTTAATTTATT

+345 CACTCGTGATATATGTTTTGACTTTTGCAGACCAGCAATGCTATTTCTGAAGCCGCTTATTCCTACAAGAAAATTGGTAT

+425 GTTGTAATTTCTATACAACATTTTGTCACCTTTTTCAATTATAAATATTTATTCCCCTATGTCCTGTATCAATTATAAAC

+505 AATACACTTTTTATATAAATATATATTAAACCTGGAACATCCTTAGTGAAATTTCTTTGTGTTGTTTATATATGTACAGA

+585 CTGTGGGGGAGCTTGTGCAGCAAGGTGCCGATTATCATCAAGGCCAAGATTGTGTAATAGGGCATGTGGAACTTGTTGTG

+665 CTAGATGCAACTGTGTTCCTCCTGGTACTTCTGGCAACACTGAGACTTGCCCTTGCTATGCCAGTTTGACTACTCATGGC

+745 AACAAACGTAAATGCCCT**TAA**TTTTCTTCTAAAATATCCTCTACCAGTATATTGTGCGATTACTATGAATATGTTATTGT

+825 TCTGTCATATATTGTGTCAATATTCAAAATCCAGTAAAATGTCTTTAGCGTGTACTTTTTTTTTGGTGGGTTGTCTTTTT

+905 AATTTTGAGGAAATTGTATTCACTTTGATTTGTGTGTTTGGTGTGATATTTGTTCTGGTTTGCATTGTATTGTATTGTAT

+985 AATAAGATGAGGAATAAATGAAGTTCGAGTATATTTTTTTATATTTGTTCTGTTCTTGGATGTTAAGTTTTATAGTACAG

+1065 AATTAAATAAAGATTATTTTATTTGTCTTATGCGCTGCTCATTTTTCATACTATTGCATTGCTTCTAATTTACTTCTATA

+1145 CACGAATGGTTTAAAAGAACTTTTATAACATCACGTCACCTGATTTAAATCTCGTGGTATTGTACATGTCAGGTAAAATG

+1225 AAAATAAAACGTTATAATAAAATATAGAAAGCGACACTTGTTAAAACAGAATAGAAAGAAAAATAAAACACCAGAAGGAA

+1305 TACTAATTAATTATCGATGAACAATCTAATTTAGATTATTATGACAAACTTCATTGCTCTTACTCAAATTATGTTATATA

+1385 GTAGACAACAAAAAGAAAAGGTTCAACAATAGGACTTGCATCGAGTATTCTTTTATCATATTCAAATAGTCAGATTCTTT

+1465 TTCGTTTTATTTTTTTCGATTTATCGAAAAATTTCCAAGGGTCAATGACGCACGATTCAAATAGTGAGATTCTTTGTAAG

+1545 AATACCCTCAAATGTAATCCCAGGACCAAATGCCAGAATCAATCCCCAATCAGTGATGTCTTTTTTCTCCTCCAACATGT

+1625 ATTCCAGCACATACACAATTGTATTGCTACTTGCATTTCCATAATCTGCCAAAGCCCTTCTGCTAGCACTCAATTTCTCA

+1705 GGAGACAAATCTAGCTTTTTTTCTAGCCTGTTTAAAATAGCAGGACCACCTGGATGAACTG
