# Supplementary material for: Structure and expression of GSL1 and GSL2 genes encoding gibberellin stimulated-like proteins in diploid and highly heterozygous tetraploid potato reveals their highly conserved and essential status
Source: BMC Genomics. 2014 Jan 2;15:2. doi: 10.1186/1471-2164-15-2 (PMC3890649; doi:10.1186/1471-2164-15-2)
Supplement: Additional file 3: Table S1 — List of GSL and GASA genes and their genetic position in potato. The chromosomal location is supported by super-scaffolds anchored via a genetic map generated for DM [54], incorporating information from RH and tomato, or a genetic map of RH generated by whole genome profiling (WGP) [55]. Locations of the GSL1 and GSL2 genes were identified by the superscaffold location on the physical map given in the agp file generated by the Potato Genome Sequencing Consortium (PGSC) (http://solanaceae.plantbiology.msu.edu/pgsc_download.shtml). The alignment of coding regions to determine identity to GSL1 and GSL2 used MUSCLE [47] and was based on allele a1 for GSL1 (FJ195646) and allele b1 for GSL2 (EU848498). [file 1471-2164-15-2-S3.pdf]

Supplementary Table 1. List of *GSL* and *GASA* genes and their genetic position in potato. The chromosomal location is supported by super-scaffolds anchored via a genetic map generated for DM, incorporating information from RH and tomato, or a genetic map of RH generated by whole genome profiling (WGP) [54,55]. Locations of the *GSL1* and *GSL2* genes were identified by the superscaffold location on the physical map given in the agp file generated by the Potato Genome Sequencing Consortium (PGSC) (http://solanaceae.plantbiology.msu.edu/pgsc_download.shtml). The alignment of coding regions to determine identity to *GSL1* and *GSL2* used MUSCLE [47] and was based on allele a1 for *GSL1* (FJ195646) and allele b1 for *GSL2* (EU848498).

Best transcript	Description	DM super-scaffold ID	PGSC Gene Id	Chromosome	Chromosome position supported by the following maps	*GSL1* exon % identity	*GSL2* exon % identity
PGSC0003DMT400040298	*GASA3*	PGSC0003DMB000000210	PGSC0003DMG400015602	5	DM, tomato	48.3	52.9
PGSC0003DMT400050285	*GASA4*	PGSC0003DMB000000497	PGSC0003DMG401019533	3	WGP, tomato	48.0	51.5
PGSC0003DMT400083077	*GASA4.1*	PGSC0003DMB000000180	PGSC0003DMG400033044	6	DM, RH, tomato	49.2	48.7
PGSC0003DMT400003089	*GASA4.2*	PGSC0003DMB000000967	PGSC0003DMG400001227	6	WGP	52.1	47.0
PGSC0003DMT400003510	*GAST1*	PGSC0003DMB000000004	PGSC0003DMG401001384	2	DM, RH, tomato	47.7	50.3
PGSC0003DMT400019726	*GAST1.1*	PGSC0003DMB000000249	PGSC0003DMG400007621	4	DM, RH	50.4	51.8
PGSC0003DMT400040585	*RSI1*	PGSC0003DMB000000505	PGSC0003DMG402015689	11	DM, RH, tomato	52.9	55.0
PGSC0003DMT400003087	*RSI1.1*	PGSC0003DMB000000967	PGSC0003DMG400001226	6	WGP	55.8	51.4
**PGSC0003DMT400055426**	***GSL1***	**PGSC0003DMB000000381**	**PGSC0003DMG400021517**	**4**	**WGP, tomato**	**98.9**	**48.6**
PGSC0003DMT400037423	*GSL1.1*	PGSC0003DMB000000078	PGSC0003DMG400014441	12	DM, RH, tomato	78.3	47.2
PGSC0003DMT400047535	*GSL1.2*	PGSC0003DMB000000341	PGSC0003DMG400018474	8	DM	73.0	49.9
PGSC0003DMT400066182	*GSL1.3*	PGSC0003DMB000000073	PGSC0003DMG400025759	1	DM, RH, tomato	67.6	44.2
**PGSC0003DMT400004046**	***GSL2***	**PGSC0003DMB000000290**	**PGSC0003DMG400001598**	**1**	**DM, RH, tomato**	**49.7**	**100**
PGSC0003DMT400009368	*GSL2.1*	PGSC0003DMB000000020	PGSC0003DMG400003642	2	DM, RH, tomato	47.3	59.9
PGSC0003DMT400009367	*GSL2.2*	PGSC0003DMB000000020	PGSC0003DMG400003641	2	DM, RH, tomato	44.1	56.2
PGSC0003DMT400075426	*GSL2.3*	PGSC0003DMB000000034	PGSC0003DMG400029338	12	DM, RH, tomato	46.7	59.6
PGSC0003DMT400062877	*GSL2.4*	PGSC0003DMB000000062	PGSC0003DMG400024474	3	DM, tomato	41.8	51.9
PGSC0003DMT400023914	*GSL2.5*	PGSC0003DMB000000133	PGSC0003DMG400009244	11	DM, RH, tomato	51.4	56.6
