# Supplementary material for: Structure and expression of GSL1 and GSL2 genes encoding gibberellin stimulated-like proteins in diploid and highly heterozygous tetraploid potato reveals their highly conserved and essential status
Source: BMC Genomics. 2014 Jan 2;15:2. doi: 10.1186/1471-2164-15-2 (PMC3890649; doi:10.1186/1471-2164-15-2)
Supplement: Additional file 4: Table S2 — SNP frequency in all GSL and GASA-like genes and a number of housekeeping genes in potato. The next generation sequence data from four tetraploid potato genotypes (‘Karaka’, ‘Summer Delight’, 1021/1, VTn62-33-3), plus the diploid RH [33], were aligned with the genome of DM [33]. Output is from polySNP tool with stringent calling (https://github.com/mfiers/polysnp). SNP frequency is given as nucleotides/SNP; ‘-‘ indicates no SNPs present. [file 1471-2164-15-2-S4.pdf]

Supplementary Table 2. SNP frequency in all GSL and GASA-like genes and a number of housekeeping genes in potato. The next generation sequence data from four tetraploid potato genotypes ('Karaka', 'Summer Delight', 1021/1, VT^{n}62-33-3), plus the diploid RH [33], were aligned with the genome of DM [33]. Output is from polySNP tool with stringent calling (https://github.com/mfiers/polysnp). SNP frequency is given as nucleotides/SNP; '-' indicates no SNPs present.

PGSC Gene ID	Name	Exon SNP frequency	Intron SNP frequency	5'UTR SNP frequency	3'UTR SNP frequency
PGSC0003DMG400018474	GSL1.2	270	46	19	23
PGSC0003DMG402015689	RSI1	97	28	27	21
PGSC0003DMG400025759	GSL1.3	90	22	63	77
PGSC0003DMG400028193	tubulin	82	28	22	34
PGSC0003DMG400018449	actin	76	38	20	105
PGSC0003DMG400029926	tubulin	75	35	-	50
PGSC0003DMG400009938	tubulin	71	39	45	-
PGSC0003DMG400007621	GAST1.1	69	32	21	15
**PGSC0003DMG400021517**	**GSL1**	**67**	**21**	**33**	**41**
PGSC0003DMG400004440	cyclophilin	58	-	-	80
PGSC0003DMG400023429	actin	57	17	18	76
PGSC0003DMG400030431	tubulin	56	27	29	24
PGSC0003DMG400019131	tubulin	53	11	27	32
**PGSC0003DMG400001598**	**GSL2**	**53**	**34**	**-**	**50**
PGSC0003DMG400029338	GSL2.3	49	33	-	44
PGSC0003DMG401001384	GAST1	46	17	68	16
PGSC0003DMG400003642	GSL2.1	44	30	-	31
PGSC0003DMG400021527	Aprt	42	29	28	22
PGSC0003DMG400023708	actin	42	33	22	58
PGSC0003DMG400027746	actin	42	22	46	38

PGSC0003DMG400020850	tubulin	41	20	47	21
PGSC0003DMG400009244	GSL2.5	40	15	34	77
PGSC0003DMG400001226	RSI1.1	36	24	40	33
PGSC0003DMG400001227	GASA4.2	33	24	32	18
PGSC0003DMG400011088	tubulin	32	25	86	22
PGSC0003DMG400024474	GSL2.4	31	25	49	18
PGSC0003DMG400033044	GASA4.1	31	25	31	26
PGSC0003DMG400008117	EF1α	31	21	25	23
PGSC0003DMG400000439	actin	28	34	34	25
PGSC0003DMG401019533	GASA4	26	20	16	50
PGSC0003DMG400023270	EF1α	25	40	14	23
PGSC0003DMG400014441	GSL1.1	24	18	23	35
PGSC0003DMG400023272	EF1α	24	40	-	27
PGSC0003DMG400015602	GASA3	23	17	17	26
PGSC0003DMG400019677	EF1α	22	16	27	27
PGSC0003DMG400003641	GSL2.2	20	20	10	19
PGSC0003DMG400001630	cyclophilin	14	-	46	26
