# Supplementary material for: Structure and expression of GSL1 and GSL2 genes encoding gibberellin stimulated-like proteins in diploid and highly heterozygous tetraploid potato reveals their highly conserved and essential status
Source: BMC Genomics. 2014 Jan 2;15:2. doi: 10.1186/1471-2164-15-2 (PMC3890649; doi:10.1186/1471-2164-15-2)
Supplement: Additional file 5: Table S3 — Motifs identified in the DM GSL1 promoter. Analysis used Genomatix-MatInspector [41] based on PLACE [42]. [file 1471-2164-15-2-S5.pdf]

Supplementary Table 3. Motifs identified in the DM *GSL1* promoter. Analysis used Genomatix-MatInspector [41] based on PLACE [42].

Related function	ID / IUPAC	Motif sequence	Organism described	Sequence	Position		Strand
					Start	End	
Light regulated	-10PEHVPSBD	TATTCT	*Hordeum vulgare*	TATTCT<br>TATTCT<br>TATTCT	-1087<br>-1046<br>-551	-1092<br>-1051<br>-556	-<br>-<br>-
Endosperm-specific expression	-300ELEMENT	TGHAAARK	*Triticum aestivum*	TGCAAAAG<br>TGTAAAGT	-1763<br>-970	-1756<br>-977	+<br>-
Endosperm-specific expression	AACACOREOSGLUB1	AACAAAC	*Oryza sativa*	AACAAAC	-1833	-1827	+
Sugar-repression (A-box/G motif)	ACGTABOX	TACGTA	*Oryza sativa*	TACGTA	-809	-804	+
Ammonium response (motif IVD)	AMMORESIVDCRNIA1	CGAACTT	*Chlamydomonas reinhardtii*	CGAACTT	-1042	-1036	+
Sugar starvation (amylase element)	AMYBOX2	TATCCAT	*Hordeum vulgare*<br>*Oryza sativa*<br>*Triticum aestivum*	TATCCAT	-1552	-1558	-
Anaerobic induction	ANAERO1CONSENSUS	AAACAAA	*Zea mays*<br>*Arabidopsis thaliana*<br>*Pisum sativum*<br>*Hordeum vulgare*<br>*Oryza sativa*<br>*Petunia hybrida*<br>*Solanum lycopersicum*	AAACAAA	-475	-469	+

Auxin responsive	ARFAT	TGTCTC	*Arabidopsis thaliana*<br>*Glycine max*<br>*Oryza sativa*	TGTCTC	-1280	-1285	-
Auxin/salicylic acid/light/ stress (ASF-1 motif)	ASF1MOTIFCAMV	TGACG	*Cauliflower mosaic virus*<br>*Nicotiana tabacum*<br>*Arabidopsis thaliana*	TGACG<br>TGACG	-1331<br>-454	-1327<br>-451	+<br>+
Phosphatase ABI1 & hormone responsive	ATHB6COREAT	CAATTATTA	*Arabidopsis thaliana*	CAATTATTA	-1172	-1164	+
Auxin responsive element	AUXREPSIAA4	KGTCCCAT	*Pisum sativum*	TGTCCCAT	-1914	-1907	+
Disease responsive	BIHD1OS	TGTCA	*Oryza sativa*	TGTCA<br>TGTCA<br>TGTCA<br>TGTCA<br>TGTCA	-1024<br>-878<br>-873<br>-385<br>-110	-1028<br>-874<br>-869<br>-389<br>-106	-<br>+<br>+<br>-<br>+
Plastid (Box II)	BOXIINTPATPB	ATAGAA	*Nicotiana tabacum*	ATAGAA	-508	-503	+
Embryo- and endosperm-specific	CANBNNAPA	CNAACAC	*Brassica napus*	CGAACAC	-1368	-1374	-
Gibberellin responsive (CARE-motif)	CAREOSREP1	CAACTC	*Oryza sativa*	CAACTC	-682	-677	+
Auxin responsive	CATATGGMSAUR	CATATG	*Glycine max*	CATATG	-1919	-1914	+
Phytochrome Myb-related transcription factor (CCA1 binding site)	CCA1ATLHCB1	AAMAATCT	*Arabidopsis thaliana*	AAAAATCT	-1943	-1936	+
Sugar starvation (CGCG element)	CGACGOSAMY3	CGACG	*Oryza sativa*	CGACG	-1549	-1545	+

Mitochondrial expression	CONSERVED11NTZMATP	ACGTATTAAAA	*Zea mays*	ACGTATTAAAA	-808	-798	+
Cytokinin responsive	CPBCSPOR	TATTAG	*Cucumis sativus*	TATTAG<br>TATTAG<br>TATTAG	-1683<br>-1528<br>-808	-1678<br>-1523<br>-813	+<br>+<br>-
Low-CO_2 response (EEC motif)	EECCRCAH1	GANTTNC	*Chlamydomonas reinhardtii*	GAGTTAC	-492	-498	-
Nitrogen response (endosperm motif)	EMHVCHORD	TGTAAAGT	*Hordeum vulgare*	TGTAAAGT	-970	-977	-
Ethylene responsive and senescence-specific (ERE)	ERELEE4	AWTTCAAA	*Solanum lycopersicum*<br>*Dianthus caryophillus*<br>*Solanum chilense*	ATTTCAAA<br>AATTCAAA<br>ATTTCAAA<br>ATTTCAAA	-1204<br>-722<br>-595<br>-139	-1197<br>-715<br>-588<br>-146	+<br>+<br>+<br>-
Light responsive	GT1CORE	GGTTAA	*Pisum sativum*	GGTTAA	-169	-174	-
Pathogen- and salt-responsive (GT-1 motif)	GT1GMSCAM4	GAAAAA	*Glycine max*	GAAAAA<br>GAAAAA<br>GAAAAA<br>GAAAAA<br>GAAAAA<br>GAAAAA	-1678<br>-1069<br>-918<br>-826<br>-706<br>-505	-1673<br>-1064<br>-923<br>-821<br>-701<br>-500	+<br>+<br>-<br>+<br>+<br>+
Light regulated (I box/I-box)	IBOX	GATAAG	*Solanum lycopersicum*<br>*Arabidopsis thaliana*	GATAAG	-537	-542	-
Light regulated (I box/I-box)	IBOXCORE	GATAA	Angiosperms	GATAA<br>GATAA<br>GATAA<br>GATAA<br>GATAA<br>GATAA	-1834<br>-1545<br>-1483<br>-1463<br>-1242<br>-1121	-1838<br>-1541<br>-1487<br>-1459<br>-1238<br>-1117	-<br>+<br>-<br>+<br>+<br>+

				GATAA<br>GATAA<br>GATAA<br>GATAA<br>GATAA	-1109<br>-834<br>-537<br>-235<br>-117	-1105<br>-830<br>-541<br>-239<br>-121	+<br>+<br>-<br>-<br>-
Low-temperature response (LTRE-1)	LTRE1HVBLT49	CCGAAA	*Hordeum vulgare*	CCGAAA	-984	-989	-
Dehydration response (MYB recognition site)	MYB1AT	WAACCA	*Arabidopsis thaliana*	AAACCA<br>AAACCA<br>TAACCA<br>AAACCA	-1927<br>-703<br>-173<br>-15	-1922<br>-698<br>-168<br>-10	+<br>+<br>+<br>+
Gibberellin responsive	MYBGAHV	TAACAAA	*Hordeum vulgare*<br>*Oryza sativa*	TAACAAA	-1101	-1095	+
Transcriptional activator (core motif of *MybSt1*, a potato MYB homolog binding site)	MYBST1	GGATA	*Solanum tuberosum*	GGATA<br>GGATA<br>GGATA<br>GGATA<br>GGATA	-1556<br>-1299<br>-1122<br>-835<br>-116	-1552<br>-1303<br>-1118<br>-831<br>-120	+<br>-<br>+<br>+<br>-
Root nodule-specific	OSE1ROOTNODULE	AAAGAT	*Vicia faba*<br>*Medicago truncatula*<br>*Glycine max*<br>*Sesbania rostrata*	AAAGAT<br>AAAGAT<br>AAAGAT<br>AAAGAT	-1764<br>-1759<br>-1220<br>-511	-1769<br>-1754<br>-1215<br>-506	-<br>+<br>+<br>+
Root nodule-specific	OSE2ROOTNODULE	CTCTT	*Vicia faba*<br>*Medicago truncatula*<br>*Glycine max*<br>*Sesbania rostrata*	CTCTT<br>CTCTT	-1205<br>-1091	-1209<br>-1095	-<br>-
Phosphate starvation	P1BS	GNATATNC	*Arabidopsis thaliana*<br>*Solanum lycopersicum*<br>*Medicago truncatula*	GGATATTC	-1556	-1549	+

			Hordeum vulgare				
Proline- & hypo-osmolarity response	PREATPRODH	ACTCAT	*Arabidopsis thaliana*	ACTCAT	-680	-675	+
Endosperm expression (Prolamine box)	PROLAMINBOXOSGLUB1	TGCAAAG	*Oryza sativa*	TGCAAAG	-1761	-1767	-
Gibberellin and abscisic acid responsive(pyrimidine box)	PYRIMIDINEBOXHVEPB	TTTTTTCC	*Hordeum vulgare*	TTTTTTCC	-820	-827	-
Gibberellin responsive and sugar repression	PYRIMIDINEBOXOSRAM	CCTTTT	*Oryza sativa*<br>*Hordeum vulgare*	CCTTTT	-826	-831	-
Rosette leaf- and root-specific	RAV1AAT	CAACA	*Arabidopsis thaliana*	CAACA<br>CAACA<br>CAACA<br>CAACA<br>CAACA<br>CAACA<br>CAACA	-1834<br>-1320<br>-1242<br>-981<br>-339<br>-281<br>-169	-1830<br>-1316<br>-1246<br>-977<br>-335<br>-285<br>-165	+<br>+<br>-<br>+<br>+<br>-<br>+
Phytochrome regulation (REalpha)	REALPHALGLHCB21	AACCAA	*Lemna gibba*	AACCAA	-167	-162	+
Abscisic acid responsive (RY repeat)	RYREPEATBNNAPA	CATGCA	*Brassica napus*	CATGCA	-1931	-1926	+
Modulation of glycinin genes	RYREPEATGMGY2	CATGCAT	*Glycine max*	CATGCAT	-1785	-1779	+

Seed-specific (legumin box)	RYREPEATLEGUMINBOX	CATGCAY	*Glycine max*	CATGCAC<br>CATGCAC	-1701<br>-1262	-1707<br>-1256	-<br>+
Plastid-specific (S1F box)	S1FBOXSORPS1L21	ATGGTA	*Spinacia oleracea*	ATGGTA	-140	-135	+
Seed development (SEF1 binding site)	SEF1MOTIF	ATATTTAWW	*Glycine max*	ATATTTATT<br>ATATTTATT<br>ATATTTAAA<br>ATATTTAAT<br>ATATTTATT	-898<br>-546<br>-467<br>-268<br>-195	-890<br>-554<br>-459<br>-260<br>-187	+<br>-<br>+<br>+<br>+
Seed development (SEF3 binding site)	SEF3MOTIFGM	AACCCA	*Glycine max*	AACCCA<br>AACCCA	-1147<br>-365	-1152<br>-360	-<br>+
Light regulated	SORLIP1AT	GCCAC	*Arabidopsis thaliana*	GCCAC	-1182	-1178	+
Light regulated	SORLIP2AT	GGGCC	*Arabidopsis thaliana*	GGGCC	-1773	-1777	-
Root tuber-specific	SP8BFIBSP8BIB	TACTATT	*Ipomoea batatas*	TACTATT<br>TACTATT	-1744<br>-1604	-1750<br>-1610	-<br>-
Axillary bud-specific (sugar-repressive element)	SREATMSD	TTATCC	*Arabidopsis thaliana*	TTATCC<br>TTATCC<br>TTATCC	-1117<br>-830<br>-121	-1122<br>-835<br>-116	-<br>-<br>+
Sulfur-responsive element	SURECOREATSULTR11	GAGAC	*Arabidopsis thaliana*	GAGAC<br>GAGAC	-1283<br>-959	-1279<br>-955	+<br>+
Enhancer (SV40 core enhancer)	SV40COREENHAN	GTGGWWHG	*Pisum sativum*<br>*Arabidopsis thaliana*	GTGGTTTG	-1921	-1928	-

Guard cell-specific	TAAAGSTKST1	TAAAG	*Solanum tuberosum*	TAAAG<br>TAAAG<br>TAAAG<br>TAAAG<br>TAAAG	-1242<br>-972<br>-905<br>-599<br>-570	-1238<br>-976<br>-909<br>-603<br>-566	+<br>-<br>-<br>-<br>+
Gibberellin responsive and sugar starvation	TATCCAOSAMY	TATCCA	*Oryza sativa*	TATCCA<br>TATCCA<br>TATCCA<br>TATCCA	-1552<br>-1302<br>-1118<br>-831	-1557<br>-1297<br>-1123<br>-836	-<br>+<br>-<br>-
Light responsive (T-box)	TBOXATGAPB	ACTTTG	*Arabidopsis thaliana*	ACTTTG<br>ACTTTG	-1410<br>-1156	-1415<br>-1151	-<br>+
Salicylic acid responsive (W-box)	WBOXATNPR1	TTGAC	*Arabidopsis thaliana*	TTGAC<br>TTGAC	-1591<br>-1021	-1587<br>-1017	+<br>+
Endosperm-specific and sugar responsive	WBOXHVISO1	TGACT	*Hordeum vulgare*	TGACT<br>TGACT	-1818<br>-272	-1822<br>-268	-<br>+
Root apical meristem-specific	WUSATAg	TTAATGG	*Oryza sativa*	TTAATGG	-15	-21	-
