# Supplementary material for: Structure and expression of GSL1 and GSL2 genes encoding gibberellin stimulated-like proteins in diploid and highly heterozygous tetraploid potato reveals their highly conserved and essential status
Source: BMC Genomics. 2014 Jan 2;15:2. doi: 10.1186/1471-2164-15-2 (PMC3890649; doi:10.1186/1471-2164-15-2)
Supplement: Additional file 6: Table S4 — Motifs identified in the DM GSL2 promoter. Analysis used Genomatix-MatInspector [41] based on PLACE [42]. [file 1471-2164-15-2-S6.pdf]

Supplementary Table 4. Motifs identified in the DM *GSL2* promoter. Analysis used Genomatix-MatInspector [41] based on PLACE [42].

Related function	ID / IUPAC	Motif sequence	Organism described	Sequence	Position		Strand
					Start	End	
Endosperm-specific (ACGT motif)	ACGTOSGLUB1	GTACGTG	*Oryza sativa*	GTACGTG	-194	-200	-
Transcriptional activator (MYB binding site)	MYBPLANT	MACCWAMC	*Antirrhinum majus*<br>*Phaseolus vulgaris*<br>*Petunia hybrida*<br>*Arabidopsis thaliana*<br>*Zea mays*<br>*Petroselinum crispum*	CACCTACC	-353	-346	+
Tissue-specific expression (RY repeat motif)	RYREPEATVFLEB4	CATGCATG	*Phaseolus vulgaris*<br>*Glycine max*<br>*Vicia faba*<br>*Oryza sativa*<br>*Arabidopsis thaliana*	CATGCATG	-182	-175	+
Circadian regulation	EVENINGAT	AAAATATCT	*Arabidopsis thaliana*<br>*Solanum melongena*	AAAATATCT	-275	-283	-
Transcriptional activator (phenylalanine ammonia-lyase gene)	BOXLCOREDCPAL	ACCWWCC	*Daucus carota*	ACCTACC	-352	-346	+
Plastid-specific (Box II)	BOXIINTPATPB	ATAGAA	*Nicotiana tabacum*	ATAGAA	-102	-107	-

Sucrose responsive element (regulation of a potato tuber storage protein)	SURE1STPAT21	AATAGAAAA	*Solanum tuberosum*	AATAGAAAA	-101	-109	-
Light regulated	SORLREP3AT	TGTATATAT	*Arabidopsis thaliana*	TGTATATAT	-160	-152	+
Light regulated	-10PEHVPSBD	TATTCT	*Hordeum vulgare*	TATTCT	-129	-124	+
Endosperm-specific	-300ELEMENT	TGHAAARK	*Triticum aestivum*	TGAAAAAT	-20	-27	-
Embryo- and endosperm-specific	CANBNNAPA	CNAACAC	*Brassica napus*	CTAACAC	-361	-367	-
Cytokinin responsive	CPBCSPOR	TATTAG	*Cucumis sativus*	TATTAG	-310	-315	-
Light regulated	GT1CORE	GGTTAA	*Pisum sativum*	GGTTAA	-222	-227	-
Pathogen- and salt-responsive (GT-1 motif)	GT1GMSCAM4	GAAAAA	*Glycine max*	GAAAAA<br>GAAAAA<br>GAAAAA<br>GAAAAA<br>GAAAAA	-273<br>-193<br>-143<br>-105<br>-21	-278<br>-188<br>-148<br>-110<br>-26	-<br>+<br>-<br>-<br>-
Light regulated(I box)	IBOXCORE	GATAA	Angiosperms	GATAA	-434	-430	+
Gibberellin responsive	MYBGAHV	TAACAAA	*Hordeum vulgare*<br>*Oryza sativa*	TAACAAA	-467	-473	-
Transcriptional activator (core motif of *MybSt1*, a potato MYB binding site)	MYBST1	GGATA	*Solanum tuberosum*	GGATA	-437	-433	+
Modulation of glycinin genes	RYREPEATGMGY2	CATGCAT	*Glycine max*	CATGCAT	-490	-496	-

Auxin induction & tissue-specific expression	NTBBF1ARROLB	ACTTTA	*Agrobacterium rhizogenes*	ACTTTA	-374	-369	+
Root nodule-specific	OSE1ROOTNODULE	AAAGAT	*Vicia faba*<br>*Medicago truncatula*<br>*Glycine max*<br>*Sesbania rostrata*	AAAGAT	-6	-1	+
Root nodule-specific	OSE2ROOTNODULE	CTCTT	*Vicia faba*<br>*Medicago truncatula*<br>*Glycine max*<br>*Sesbania rostrata*	CTCTT<br>CTCTT<br>CTCTT<br>CTCTT	-553<br>-510<br>-449<br>-450	-549<br>-514<br>-503<br>-446	+<br>-<br>-<br>+
Phytochrome regulation (REalpha)	REALPHALGLHCB21	AACCAA	*Lemna gibba*	AACCAA	-412	-407	+
Plastid-specific (S1F box)	S1FBOXSORPS1L21	ATGGTA	*Spinacia oleracea*	ATGGTA	-488	-483	+
Axillary bud-specific (sugar-repressive element)	SREATMSD	TTATCC	*Arabidopsis thaliana*	TTATCC	-432	-437	-
Guard cell-specific	TAAAGSTKST1	TAAAG	*Solanum tuberosum*	TAAAG	-369	-373	-
Light regulated (T-box)	TBOXATGAPB	ACTTTG	*Arabidopsis thaliana*	ACTTTG	-330	-325	+
Sugar responsive	WBOXHVISO1	TGACT	*Hordeum vulgare*	TGACT	-13	-17	-
Root apical meristem-specific	WUSATAg	TTAATGG	*Oryza sativa*	TTAATGG	-224	-230	-
