# Supplementary material for: Structure and expression of GSL1 and GSL2 genes encoding gibberellin stimulated-like proteins in diploid and highly heterozygous tetraploid potato reveals their highly conserved and essential status
Source: BMC Genomics. 2014 Jan 2;15:2. doi: 10.1186/1471-2164-15-2 (PMC3890649; doi:10.1186/1471-2164-15-2)
Supplement: Additional file 7: Table S5 — Transformation of potato antisense constructs of the GSL1 and GSL2 genes. Results are presented for three independent Agrobacterium-mediated transformation experiments of potato cultivar Iwa using the binary vectors pMOA33-Lhca3-antiGSL1 (Additional file 10: Figure S4B) and pMOA33-Lhca3-antiGSL2 (Additional file 10: Figure S4C). [file 1471-2164-15-2-S7.pdf]

Supplementary Table 5. Transformation of potato antisense constructs of the GSL1 and GSL2 genes. Results are presented for three independent *Agrobacterium*-mediated transformation experiments of potato cultivar Iwa using the binary vectors pMOA33-Lhca3-antiGSL1 (Supplementary Figure 4B) and pMOA33-Lhca3-antiGSL2 (Supplementary Figure 4C).

**Binary vector**	**Experiment**	**Number of leaf explants**	**Number of cell colonies recovered**	**Number of cell colonies regenerating shoots**
pMOA33-Lhca3-antiGSL1	1	112	10	0
	2	109	8	0
	3	132	15	0
pMOA33-Lhca3-antiGSL2	1	118	15	0
	2	121	20	0
	3	103	14	0
