# Supplementary material for: Structure and expression of GSL1 and GSL2 genes encoding gibberellin stimulated-like proteins in diploid and highly heterozygous tetraploid potato reveals their highly conserved and essential status
Source: BMC Genomics. 2014 Jan 2;15:2. doi: 10.1186/1471-2164-15-2 (PMC3890649; doi:10.1186/1471-2164-15-2)

Supplementary Figure 3. Senescing potato cell colonies transformed with antisense constructs of the GSL1 gene. Identical results were obtained for the antisense construct of the GSL2 gene.

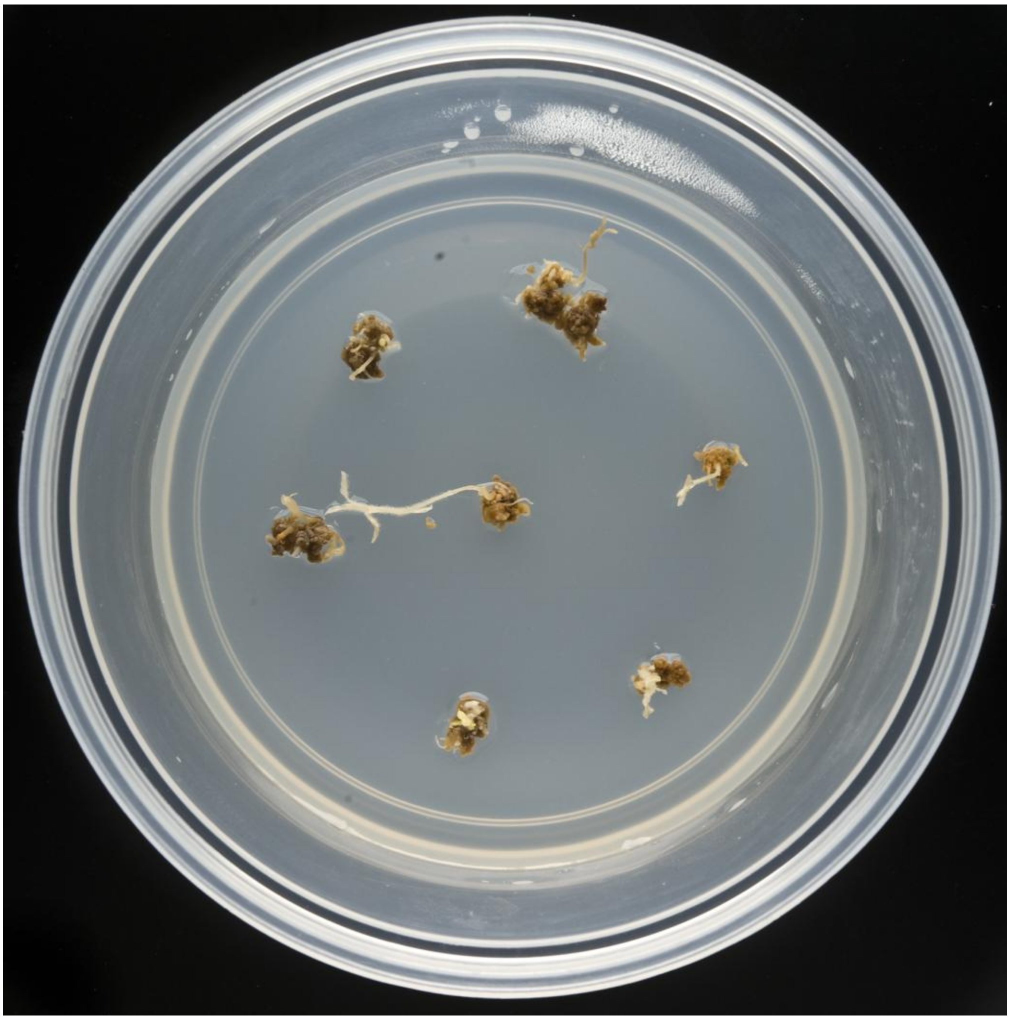

Supplement: Additional file 8: Figure S3 — Senescing potato cell colonies transformed with antisense constructs of the GSL1 gene. Identical results were obtained for the antisense construct of the GSL2 gene. [file 1471-2164-15-2-S8.pdf]
