# Supplementary material for: Structure and expression of GSL1 and GSL2 genes encoding gibberellin stimulated-like proteins in diploid and highly heterozygous tetraploid potato reveals their highly conserved and essential status
Source: BMC Genomics. 2014 Jan 2;15:2. doi: 10.1186/1471-2164-15-2 (PMC3890649; doi:10.1186/1471-2164-15-2)

Supplementary Figure 4. PCR confirmation of transgenic status of potato cell colonies transformed with antisense constructs of the GSL1 and GSL2 genes.

(A) PCR analysis of representative *Lhca3-antiGSL1* cell colonies. Products of DNA amplification after PCR using DNA from potato cell colonies as the template with primers specific to *Lhca3-antiGSL1* chimeric gene. Predicted amplicon size 840bp. Lane 1 Bioline HyperLadder II; lane 2-5 colonies A1-A4; lane 6 pMOA33-Lhca3-antiGSL1 plasmid (+); lane 7 non-transgenic potato (Iwa).

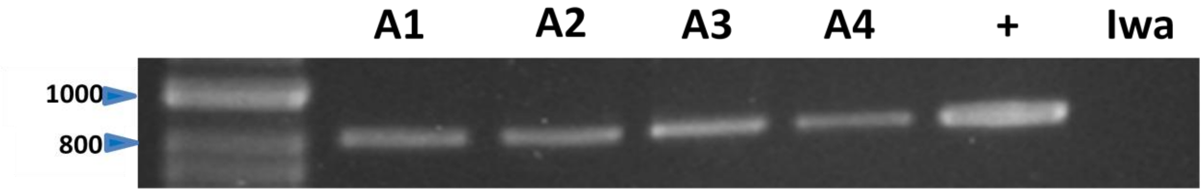

(B) PCR analysis of representative *Lhca3-antiGSL2* cell colonies. Products of DNA amplification after PCR using DNA from potato cell colonies as the template with primers specific to *Lhca3-antiGSL2* chimeric gene. Predicted amplicon size 940bp. Lane 1 Bioline HyperLadder II; lane 2-4 colonies B1-B3; lane 5 non-transgenic potato (Iwa); lane 6 pMOA33-Lhca3-antiGSL1 plasmid (+).

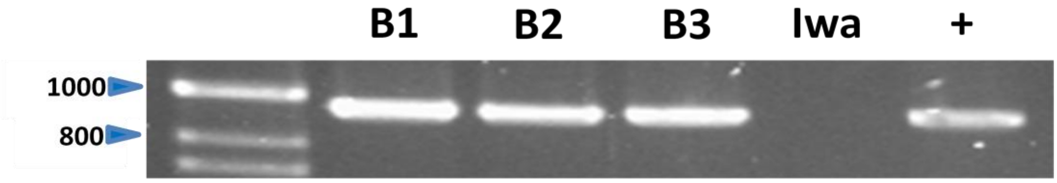

Supplement: Additional file 9: Figure S4 — PCR confirmation of transgenic status of potato cell colonies transformed with antisense constructs of the GSL1 and GSL2 genes. [file 1471-2164-15-2-S9.pdf]
