# Supplementary figures and images for: Structure and expression of GSL1 and GSL2 genes encoding gibberellin stimulated-like proteins in diploid and highly heterozygous tetraploid potato reveals their highly conserved and essential status
Source: BMC Genomics. 2014 Jan 2;15:2. doi: 10.1186/1471-2164-15-2 (PMC3890649; doi:10.1186/1471-2164-15-2)

**A**

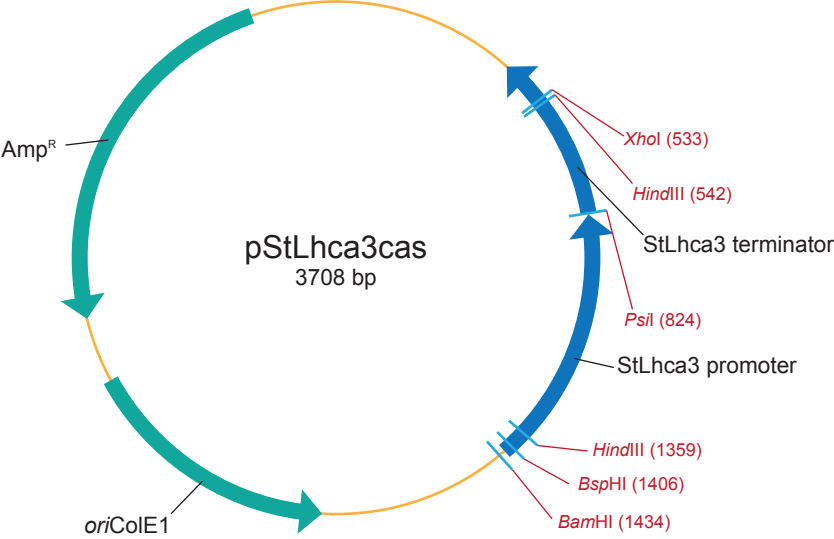

**B**

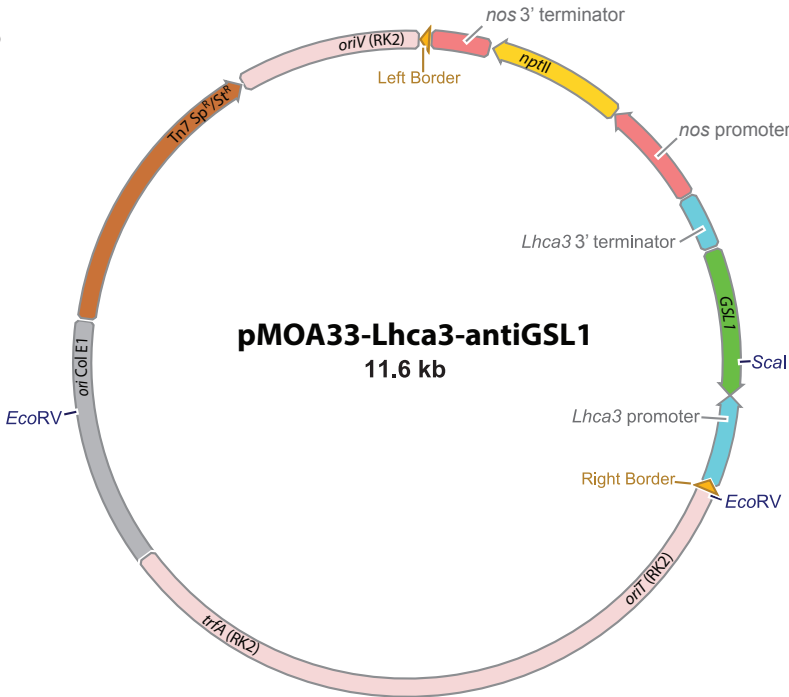

**C**

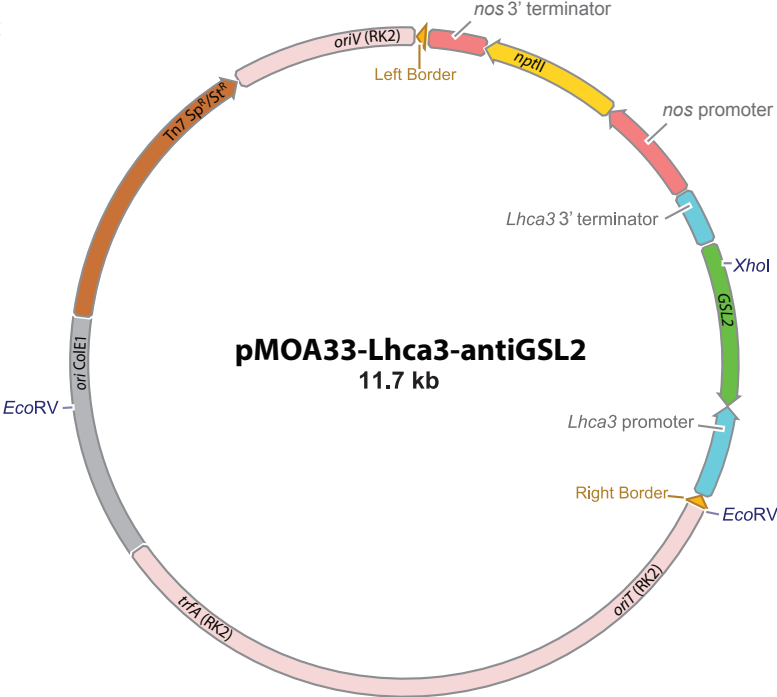

Supplement: Additional file 10: Figure S5 — Plasmids constructed and used in this study. A. pStLhca3cas; B. pMOA33-Lhca3-antiGSL1; C. pMOA33-Lhca3-antiGSL2. [file 1471-2164-15-2-S10.pdf]
